# Vector competence of lambda-cyhalothrin resistant *Aedes aegypti* strains for dengue-2, Zika and chikungunya viruses in Colombia

**DOI:** 10.1371/journal.pone.0276493

**Published:** 2022-10-25

**Authors:** Idalba M. Serrato, Diana Moreno-Aguilera, Paola A. Caicedo, Yenifer Orobio, Clara B. Ocampo, Ronald Maestre-Serrano, Dioselina Peláez-Carvajal, Martha L. Ahumada

**Affiliations:** 1 Grupo de Entomología, Instituto Nacional de Salud, Bogotá, D.C., Colombia; 2 Fundación Salutia, Bogotá, D.C., Colombia; 3 Natural Science Faculty, Universidad Icesi, Cali, Valle del Cauca, Colombia; 4 Epidemiology and Biostatistics Unit, Centro Internacional de Entrenamiento e Investigaciones Médicas-CIDEIM, Cali, Colombia; 5 Vector, Biology and Control Unit. Centro Internacional de Entrenamiento e Investigaciones Médicas-CIDEIM, Cali, Colombia; 6 Dirección de Vocaciones y Formación, Ministerio de Ciencia y Tecnología e Innovación, Minciencias, Bogotá, D.C., Colombia; 7 Facultad de Ciencias de la Salud, Universidad Simón Bolívar, Barranquilla, Atlántico, Colombia; 8 Grupo de Virología, Instituto Nacional de Salud, Bogotá, D.C. Colombia; CEA, FRANCE

## Abstract

*Aedes aegypti* is the primary vector of dengue, Zika, and chikungunya viruses. Studies have shown that insecticide resistance affects vector competence (VC) of some mosquito species. This study evaluates the effect of resistance to lambda-cyhalothrin and *kdr* V1016I mutation genotypes on the VC of *Ae*. *aegypti* strains for DENV-2, ZIKV, and CHIKV. Three *Ae*. *aegypti* strains with gradual lambda-cyhalothrin resistance (susceptible, resistant, and highly resistant) were infected with DENV-2, ZIKV, and CHIKV. Individual mosquitoes were tested to detect virus infection in the abdomen and head-salivary glands, using RT-PCR, and genotypes for V1016I mutations using allele-specific PCR. Recorded VC variables were midgut infection rate (MIR), dissemination rate (DIR), and dissemination efficiency (DIE). Lambda-cyhalothrin resistance affects differentially VC variables for ZIKV, DENV-2, and CHIKV. For ZIKV, an apparent gradual increase in DIR and DIE with the increase in insecticide resistance was observed. For DENV-2 the MIR and DIE were higher in insecticide resistant strains. For CHIKV, only MIR could be evaluated, this variable was higher in insecticide resistance strains. The presence of *kdr* V1016I mutation on mosquito resistant strains did not affect VC variables for three study viruses.

## Introduction

*Aedes aegypti* has adapted to life in urban areas where it feeds exclusively on human hosts, mainly indoors. This behavior makes *Ae*. *aegypti* is an extremely efficient vector for arboviruses, among these are dengue (DENV), chikungunya (CHIKV), and Zika (ZIKV) viruses [1]. This mosquito species is responsible for DENV transmission in tropical and subtropical regions worldwide [2]. Approximately 390 million dengue infections are reported per year, and 3.9 billion people are at risk of acquiring the infection in over 125 countries [3–5]. CHIKV has spread to over 60 countries in Asia, Africa, Europe, and America since 2004 [6]. In America, between 2018 and 2021, 237,925 confirmed cases were reported [7]. Outbreaks of ZIKV have been recorded since 2007 in 87 countries and territories of Africa, America, Asia, and the Pacific islands [8]. In America, between 2015 and 2021, 890,933 confirmed cases were reported [9].

In Colombia, dengue is considered a public health priority due to its endemic transmission, with confirmed cocirculation of the four serotypes. A total of 1.4 million dengue cases were reported between 1990 and 2016 [10], and the largest epidemic in recent years was reported in 2019–2020, with an incidence of 475.4 cases/100,000 inhabitants [11]. Chikungunya and Zika were introduced into the country in 2014 and 2015, respectively, causing soon after epidemic outbreaks [10]. For chikungunya, 359,728 cases were reported in 2015 [12], and since this date, a significant reduction, from 19,566 cases in 2016 to 160 cases in 2020, has been reported [13,14]. Regarding Zika, 96,860 cases were reported in 2016 [13], with a similar pattern to chikungunya since the outbreak, from 2,054 cases in 2017 to 165 cases in 2020 [14,15].

The lack of efficient and specific vaccines and medical treatments for the diseases caused by these viruses [16–18] has meant that prevention is focused mainly on vector control strategies to reduce larva and adult mosquito densities. In Colombia, vector control programs for *Ae*. *aegypti*, have been focused on both adulticides and larvicides. For adult control, the main insecticides have been the organophosphates, malathion, pirimiphos-methyl, and fenitrothion, and the pyrethroids, deltamethrin, and lambda-cyhalothrin, and for larval control, the organophosphorus temephos and insect growth regulators [19–23]. In the country, the continuous use of organophosphates and pyrethroids started in the 1970s and 1990s, respectively, leading to an increase in selection pressure that has contributed to the widespread of *Ae*. *aegypti* resistant populations in Colombia [19,21,23–25].

In the country, in general, pyrethroids have been one of the insecticides that have caused resistance, widely distributed in *Ae*. *aegypti* [26]. Lambda-cyhalothrin has been among the most used insecticide molecules against this mosquito species since 1993 when DDT use was banned [27]. Between 2006 and 2019, ten studies on lambda-cyhalothrin resistance for 126 *Ae*. *aegypti* populations were carried out in Colombia showing that 76% of the mosquito populations were resistant to this pyrethroid [19–21,23,25,26,28–31]. Lambda-cyhalothrin resistance in Colombia, for *Ae*. *aegypti* is associated with the presence of the V1016I and F1534C *kdr* mutations, but metabolic detoxification mechanisms also have been reported [20,21,25,31–37]. In addition, a significant correlation has been observed between the frequency I1016 genotype and 50% lethal concentration for lambda-cyhalothrin [36].

Some studies have shown that the presence of mutations associated with insecticide resistance can modify the interactions between mosquito vectors of human diseases and the pathogens that they transmit [38–40], including VC. To our knowledge, the association between insecticide resistance mutations and, in general, insecticide resistance and VC of *Ae*. *aegypti* for arbovirus have been scarcely studied. A recent study showed that when a selected permethrin-resistant population of *Ae*. *aegypti* were infected with DENV-1, there was an increase in the dissemination rate compared with a less permethrin-resistant population, suggesting an increase in VC [41]. The results also show that the permethrin-resistant population increased in the frequency of the resistance allele of *kdr* mutations V1016I and F1534C, which might explain the increase in the dissemination rate for DENV-1. Concerning other mosquitos, a study on VC in two *Culex quinquefasciatus* strains carrying Ester^2^ and *ace-1* G119S insecticide resistance mutations, respectively, for transmission of the West Nile virus (WNV) showed that the presence of the mutations significantly increases the dissemination rate and transmission efficiency of this virus in the resistant mosquito strains [39]. It is interesting to note that insecticide resistance showed similar effects on VC for viruses, with an increase in the dissemination rate but without any effect on the infection rate. In general, insecticide resistance and its associated mutations could increase the proportion of infected mosquitoes in contact with the human population and therefore the risk for disease transmission [38,39,42].

Taking into account the poor knowledge of the association between insecticide resistance and VC of *Ae*. *aegypti* for arbovirus, the present study aimed to evaluate the effect of resistance to lambda-cyhalothrin and the *kdr* V1016I and F1534C mutation genotypes on VC variables (midgut infection rate, MIR; dissemination rate, DIR, and dissemination efficiency, DIE) of three *Ae*. *aegypti* strains for DENV-2, ZIKV and, CHIKV. Our study provides evidence that insecticide resistance in *Ae*. *aegypti* has a differential effect on VC according to the virus. For ZIKV, lambda-cyhalothrin resistance increased virus dissemination, with an apparent direct correlation between these variables. For DENV-2 and CHIKV there was some evidence that insecticide resistance increased virus infection, and in addition virus dissemination for DENV-2.

## Materials and methods

### Mosquito strains

The *Ae*. *aegypti* strains included in this study were selected based on previous bioassays carried out by National Insecticide Resistance Surveillance Network at Instituto Nacional de Salud (INS), and Centro Internacional de Entrenamiento e Investigaciones Médicas (CIDEIM) that reported possible pyrethroid resistant in mosquitoes collected from localities of Casanare and Meta departments. The two field mosquito strains of *Ae*. *aegypti* used for the bioassays were collected in Nunchia municipality of Casanare Department (5°38′09″N 72°11′42″O) and Villavicencio city of Meta Department (4°15´32″N 73°62´15″O) in Colombia during 2017 and 2018, respectively. Immature stages were submitted to the Laboratorio de Entomología at INS and CIDEIM. Larvae, pupae, and adults were reared under controlled conditions at 27 ± 2°C and 60 ± 10% relative humidity. Larvae were fed with powdered cat food and adults with 10% sucrose solution *ad libitum*, and blood feeding was performed through an artificial membrane feeder using defibrinated rabbit blood to obtain filial generations. As a control strain, both for insecticide resistance and VC, a laboratory selected strain originating from Cali city (Cali-S) was selected. This strain has been maintained without exposure to insecticides for close to 50 filial generations and has been widely studied and validated as a model strain susceptible to infection for the four DENV serotypes by CIDEIM [43,44]. Adult mosquitos of this strain were reared under similar laboratory conditions to those of the resistant strains. The three selected strains were used for insecticide resistance, genotype determination, and VC bioassays.

### CDC bioassays to confirm lambda-cyhalothrin resistance of *Ae*. *aegypti* strains

Lambda-cyhalothrin resistance in *Ae*. *aegypti* strains included in the VC bioassays was confirmed using the CDC bottle bioassay [45]. Each test consisted of four insecticide treatment bottles and one control bottle. Treatment bottles were impregnated with the discriminant concentration of lambda-cyhalothrin, 10 μg/mL (technical grade 99.5; Chem Services, West Chester, PA), and the control bottle was treated with 1 mL absolute ethanol. The Cali-S, Nunchia, and Villavicencio mosquito strains were subsequently exposed, 25 female mosquitoes, 3 to 4 days old, inside the treated bottles. After 30 min of exposure, mosquitoes were recorded as dead or alive. A dead mosquito was a mosquito that could not stand and remained immobile. The results were expressed as percent mortality. According to the mortality percentage, for the discriminant concentration of lambda-cyhalothrin, the *Ae*. *aegypti* strains were classified as susceptible (≥ 98% mortality), resistant (97% - 50% mortality), and highly resistant (≤ 50% mortality). This classification is not equivalent to the intensity of resistance established by WHO, which includes five and 10 times discriminating concentrations of insecticide in the bioassays to define the resistant classification [46].

### Virus strains

Three virus strains isolated from an acute febrile patient in the Laboratorio de Virología of the Instituto Nacional de Salud were evaluated: (1) DENV-2, isolated in 2013 in Meta Department and corresponding to the genotype Asian American [47]; (2) ZIKV, isolated in 2016 in Meta Department, identified as a strain circulating in the Americas [48]; and (3) CHIKV, isolated in 2014 in the Cordoba Department and identified as an Asian genotype [49]. All viruses were inoculated in C6/36 HT cell culture (*Aedes albopictus* cells). Infected cells were incubated for 14 days for DENV-2 and CHIKV and 10 days for ZIKV at 28±1°C in Leibovitz-15 medium supplemented with 2% fetal bovine serum, 2% tryptose phosphate, 1% penicillin/streptomycin, and 1% L-glutamine [50]. Viral growth was assessed in terms of cytopathic effects induced in cell culture. Supernatants were collected in 15 mL conical centrifuge tubes and used to feed mosquitos.

### Vector competence bioassays

The analysis of vector competence was performed using three variables: midgut infection rate (MIR), dissemination rate (DIR), and dissemination efficiency (DIE). For each of the three mosquito strains with gradual lambda-cyhalothrin resistance: (1) susceptible, Cali-S, F49-F51 generations, (2) resistant, Nunchia, F3 generation, and (3) highly resistant, Villavicencio, F3-F4 generations, 100 to 200 *Ae*. *aegypti* female mosquitos, 8 to 10 days old, were exposed to an infectious blood meal containing defibrinated rabbit blood and virus supernatants of each virus strain (1:1). The titers of each infectious blood meal were DENV-2, 5,0X10^6^ (95% confidence interval (CI): 4.5X10^6^-5.4X10^6^); for ZIKV, 9.0X10^10^ (95% CI: 3.2X10^10^-1.5X10^11^); and for CHIKV 1.4X10^9^ (95% CI: 3.7X10^8^-2,4X10^9^) genome copy equivalents per milliliter (GCE/mL) (S1 Table). These values are close to the previously reported ranges in viremic patients for DENV [51–55], ZIKV [56–58], and CHIKV [52,59–61]. These titers of viral RNA were measured as explained later (Section on viral RNA absolute quantification in the infected blood-meal). One hour after feeding, fully engorged females were isolated in cages, fed *ad libitum* with 10% sucrose in water, and maintained at 27 ± 2°C and 60 ± 10% relative humidity. Fourteen days postinfection (dpi), survivor mosquitoes were sacrificed by freezing and preserved at -70°C until processing.

Each preserved mosquito was dissected under a microscope to obtain two samples: the head and salivary glands extracted from the thorax (hereafter referred to as head-salivary glands); and the abdomen, including the midgut (hereafter referred to as midgut). These samples were preserved in TRIzolTM Reagent at -70°C for subsequent RNA extractions and virus detection (Section, detection of viral infection in the mosquitos). Positive infections of the virus were first determined in the midgut. When the midgut was positive, the respective head-salivary glands were analyzed for virus infection. Based on these results, vector competence variables were estimated as follows: (1) MIR was calculated as the number of mosquitoes with viral infections in the midgut divided by the total number of surviving mosquitoes; (2) DIR was calculated as the number of mosquitoes with viral infections in the head-salivary glands divided by the number of mosquitoes with viral infections in the midgut; and (3) DIE was calculated as the number of mosquitoes with viral infections in the head-salivary glands divided by the total number of surviving mosquitoes. The assays were replicated four times.

### Detection of viral infection in the mosquitos

DENV-2, ZIKV, and CHIKV infections in the midgut and head-salivary gland were detected using real-time RT-PCR. This method is highly sensitive to detecting the presence/absence of the viral particles in different mosquito tissues allowing us to estimate the three study VC variables. Initially, RNA was extracted from the midgut and head-salivary glands tissues using TRIzolTM Reagent (Thermo Fisher Scientific, USA) according to the manufacturer’s recommendations. RNA extracted was resuspended in RNase-free water, quantified in a NanoDrop 2000^®^ spectrophotometer, and stored at -70°C. The reactions were performed in a Bio-Rad C1000 CFX96 Real-Time System thermocycler using a Luna® One-Step RT–PCR Kit E3005 by New England BioLabs Inc., USA. The final reaction volume was 10 μL, and each reaction contained 5 μL of Luna Universal One-Step Reaction Mix, 0.5 μL Luna WarmStart® RT Enzyme Mix, 0.4 μL of each specific primer [10 μM], 2.7 μL of water, and 1 μL of RNA [56,62,63]. The thermic profile reactions for DENV-2, ZIKV, and CHIKV amplification are described in the S2 Table. Positive infections were determined as follows: (1) DENV-2, Ct<37 and peak melting curve between 83.5°C and 84.5°C; (2) ZIKV, Ct<37 and peak melting curve between 80°C and 84.5°C; and (3) CHIKV, Ct<39 and peak melting curve between 83°C and 83.5°C. Different results were considered negative.

### Viral RNA absolute quantification in the infected blood-meal

Quantification of viral RNA copy numbers in mixed blood and viruses used in mosquito feeding was performed using real-time RT–qPCR and the standard curve method (S1 Table). RNA was extracted from a mixture of blood and virus using TRIzolTM Reagent (Thermo Fisher Scientific, USA) according to the manufacturer’s recommendations and stored at -70°C.

The DNA construct Mpx-CHIKV-DENV-ZIKV (T7-SP6) was used to obtain a control RNA by in vitro transcription using a HiScribe™ T7 High Yield RNA Synthesis Kit [64]. RNA control was quantified in a NanoDrop 2000^®^ spectrophotometer. Then, 10-fold serial dilutions of the control RNA were used as a standard in all RT–qPCRs. RT–qPCR for DENV-2, ZIKV, and CHIKV detection and quantification was carried out following the manufacturer’s recommendations of the Luna Universal Probe One-Step RT–qPCR kit (NEB) and virus-specific primers/probes of the previously described Mpx-CHIKV-DENV-ZIKV [64]. For each serial dilution point and RNA-extracted sample, triplicate measurements were performed. The viral concentration was expressed as genome copy equivalents per milliliter (GCE/mL).

### Genotypes and allelic frequencies of *kdr* mutations V1016I and F1534C

DNA of the mosquitoes used for the lambda-cyhalothrin resistance and VC bioassays was obtained from the thorax using a modification of the grinding buffer method (0.1 M NaCl, 0.2 sucrose, 0.1 M Tris HCl pH = 9.1–9.2, 0.05 M EDTA, 0.5% SDS) [65]. The precipitate was suspended in double-distilled water, and DNA was quantified using a NanoDrop 2000^®^ spectrophotometer and stored at -70°C.

Genotypes at the alleles V1016I and F1534C SNP loci were detected using allele-specific PCR methodology [66,67]. For locus 1016, each reaction mixture contained 10 μL of SYBR^®^ Green PCR Master Mix (Applied Biosystems 4309155), 0.2 μL of each primer (S2 Table) (100 picomoles/μL), 7.6 μL of double-distilled water, and 2 μL of DNA for a final reaction volume of 20 μL. The thermic profile reactions for amplification of the V1016I mutation are described in S2 Table. Melting curves were interpreted as follows: (1) peak at 87°C corresponded to the wild-type (homozygous dominant) VV_1016_ genotype; (2) peak at 78°C corresponded to the mutant (homozygous recessive) II_1016_ genotype; and (3) simultaneous presence of before mentioned two peaks corresponded to a heterozygous genotype VI_1016_. For locus 1534, the total volume reaction mixture was 20 μL, containing 2 μL of DNA, 10 μL of SYBR^®^ Green PCR Master Mix (Applied Biosystems 4309155), 0.066 μL of Cys1534f primer (S2 Table), 0.2 μL of Phe 1534f and 1534r (50 picomoles/μL) and 7.7 μL of double-distilled water. The thermic profile reactions for amplification of the F1534C mutation are described in S2 Table. Melting curves were interpreted as follows: (1) peak at 83°C corresponded to the homozygous wild-type (homozygous dominant) FF_1534_ genotype; (2) peak at 79°C corresponded to the mutant (homozygous recessive) CC_1534_ genotype; and (3) simultaneous presence of before mentioned two peaks corresponded to a heterozygous genotype FC_1534_.

Genotype frequencies for each mosquito strain were calculated as the proportion of mosquitoes with each genotype (VV_1016_, II_1016_, VI_1016_, FF_1534_, CC_1534,_ and FI_1534_) over the total number of mosquitoes evaluated for each V1016I and F1534C mutation. The allele frequencies (V_1016_, I_1016_, F_1534,_ and C_1534_) were calculated as twice the number of homozygotes plus the number of heterozygotes, over twice the total number of mosquitoes evaluated in each mutation for each strain.

### Statistical analysis

All vector competence variables (MIR, DIR, and DIE), as well as mortality for insecticide resistance bioassays, were expressed as percentages with their corresponding 95% confidence interval. Logistic regression models were used to determine the effect of two independent variables: (1) lambda-cyhalothrin resistant *Ae*. *aegypti* strains and (2) *kdr* mutations genotypes, on three dependent variables related to VC for DENV-2, ZIKV, and CHIKV. Lambda-cyhalothrin resistant *Ae*. *aegypti* strain was analyzed using three categories: (1) susceptible strain (Cali-S), (2) resistant strain (Nunchia), and (3) highly resistant strain (Villavicencio). For *kdr* mutation (V1016I and F1534C *kdr* mutations) genotypes, three categories were included: (1) wild-type (dominant homozygous), (2) heterozygous, and (3) mutant (homozygous recessive). The three dependent variables were: (1) midgut infection rate (MIR), (2) dissemination rate (DIR), and (3) dissemination efficiency (DIE), each treated as a binary variable (virus infection: yes or not). In the analyses, a separate model was performed for each virus. In general, the threshold of significance (α) was established at a value of 0.05. The goodness of fit of the models was evaluated with the chi-square statistic of the likelihood-ratio test. Post-hoc pairwise comparisons were performed using the Bonferroni test. All statistical analyses were performed using STATA SE 16 software. To verify whether the genotype frequencies of the *kdr* mutations V1016I and F1534C agreed with the Hardy-Weinberg equilibrium principle, Genepop software V.4.7.5 was used [68].

### Ethics statement

The study was conducted according to the guidelines of the Declaration of Helsinki and approved by the Animal Ethics Committee of the Universidad ICESI-CIECUAE (Protocol code 0018 of 2018) and Comité de Ética y Metodologías de la Investigación—CEMIN of the Instituto Nacional de Salud of Colombia (Protocol code CTIN 7–2016).

## Results

### Lambda-cyhalothrin resistance and genotype *kdr* mutations V1016I and F1534C of *Ae*. *aegypti* strains

The lambda-cyhalothrin resistance and the presence of the *kdr* mutations V1016I and F1534C in *Ae*. *aegypti* tested strains were confirmed. Using the discriminating concentration for lambda-cyhalothrin the *Ae*. *aegypti* strains were classified into three categories of gradual resistance: (1) susceptible, assigned to Cali-S strain that showed 100% (96% - 100%) mortality, (2) resistant, assigned to Nunchia strain, 85% (78% - 90%) mortality, and (3) highly resistant, assigned to Villavicencio strain, 38% (29% - 48%) mortality (Table 1). Regarding the *kdr* mutation V1016I, the genotype II_1016_ (homozygous recessive) associated with lambda-cyhalothrin resistance was more prevalent in the highly resistant strain, 0.88, followed by the resistant strain, 0.49, and this genotype was not present in the susceptible strain. On the other hand, for the *kdr* mutation F1534C, the genotype CC_1534_ (homozygous recessive), also associated with resistance in *Ae*. *aegypti*, was the most prevalent in all mosquito strains, with frequencies ranging between 0.89 and 1 (Table 1).

**Table 1:** Mortality of *Aedes aegypti* strains exposed to lambda-cyhalothrin (discriminating concentration: 10 μg/bottle by 30 min exposure to insecticide) and their genotype frequencies of *kdr* mutations V1016I and F1534C.

Strain	n	Mortality^a^ (%)	Genotype frequencies					
			Locus					
			1016			1534		
			Wild-type^b^	Heterozygous^c^	Mutant^d^	Wild-type^b^	Heterozygous^c^	Mutant^d^
Susceptible	100	100	0.99	0.01	0	0	0	1
Resistant	148	85	0.29	0.22	0.49	0.02	0.09	0.89
Highly resistant	100	38	0.06	0.06	0.88	0	0	1

^a^Mortality is calculated as the number of dead mosquitoes/total number of evaluated mosquitoes x 100.

^b^Homozygous dominant

^c^Heterozygous

^d^Homozygous recessive.

### Vector competence of the three *Ae*. *aegypti* mosquito strains with lambda-cyhalothrin resistance for DENV-2, ZIKV, and CHIKV

To determine the effect of the resistance to lambda-cyhalothrin on VC of *Ae*. *aegypti*, for three studies viruses, 35 bioassays (DENV-2 = 14, ZIKV = 10, and CHIKV = 11) with a total of 857 survivor mosquitoes (DENV-2 = 280, ZIKV = 314, and CHIKV = 263), were performed. 14 days postinfection virus infections were detected in the mosquito samples to calculate three VC variables, MIR, DIR, and DIE.

### Vector competence for DENV-2

**Fig 1:**
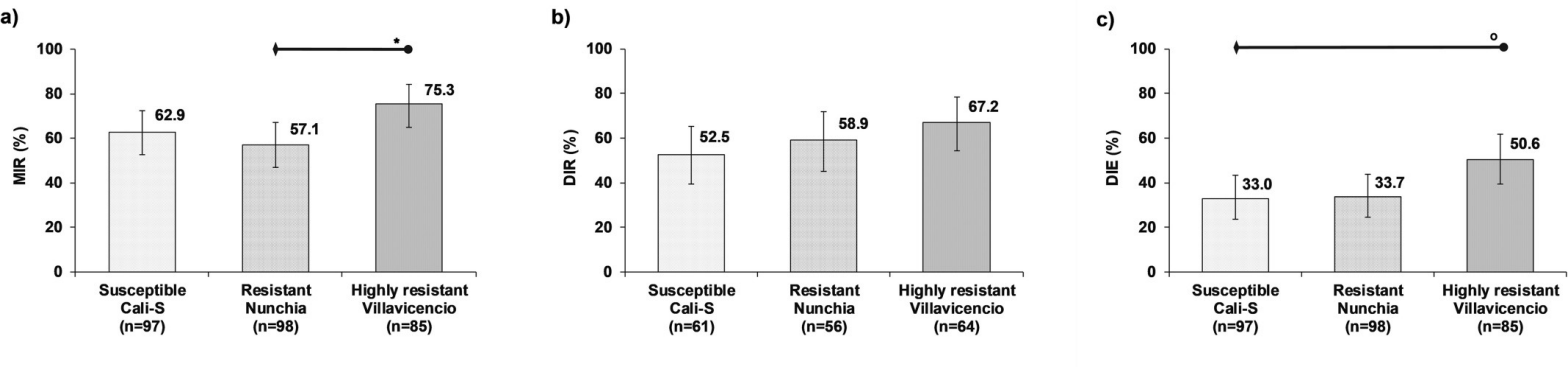
Effect of lambda-cyhalothrin resistant *Ae*. *aegypti* strains on VC for DENV-2. a) Midgut infection rate (MIR), b) Dissemination rate (DIR), and c) Dissemination efficiency (DIE). The error bars correspond to 95% confidence interval. The horizontal bars represent multiple comparisons (Bonferroni method); diamonds are the reference categories, and circles are the compare categories. Statistical significance are showed as follow: * *p*< 0.05, ** *p*< 0.01, *** *p*< 0.001, ^**o**^ *p* = 0.05 (borderline significance).

For DENV-2, the highly resistant strain showed higher MIR, DIR, and DIE, compared with resistant and susceptible strains (Fig 1). The MIR for lambda-cyhalothrin resistant mosquito strains showed a mild increase between susceptible, 62.9%, and highly resistant, 75.3%, strains (Fig 1A). The regression model showed that the MIR was influenced by mosquito strains (χ^2^_(2)_ = 6.63, *p* = 0.036). Multiple comparisons showed that the MIR for the highly resistant strain, 75.3%, was significantly higher compared with the resistant strain, 57.1% (*z* = 2.55; *p* = 0.032) (Fig 1A). However, no statistically significant differences were found between the susceptible strain and the two resistant strains (S3 Table). DIR, also showed a barely increase from 52.5% in the susceptible to 67.2% in the highly resistant strain (Fig 1B), without statistically significant differences between mosquito strains (χ^2^_(2)_ = 2.81, *p* = 0.246) (S3 Table). Finally, DIE showed the same pattern as MIR and DIR. The DIE varied from 33.0% in the susceptible strain to 50.6% in the highly resistant strain. The regression model showed that the DIE was affected by mosquito strains (χ^2^_(2)_ = 7.33, *p* = 0.026) (S3 Table). The higher DIE of the highly resistant strain compared with the susceptible strain, 50.6%, and 33.0%, respectively, was on the borderline of statistical significance (*z* = 2.39; *p* = 0.050). While, no statistically significant differences were found between the resistant strain and susceptible strain, nor between the two resistant strains (Fig 1C; S3 Table).

#### Vector competence for ZIKV

**Fig 2:**
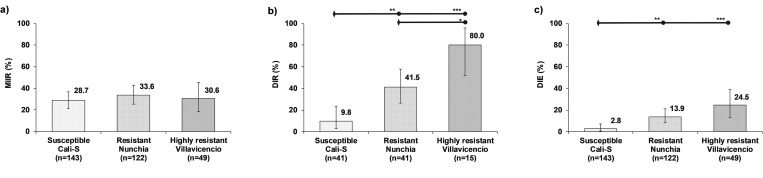
Effect of gradual lambda-cyhalothrin resistant *Ae*. *aegypti* strains on VC for ZIKV. a) Midgut infection rate (MIR), b) Dissemination rate (DIR), and c) Dissemination efficiency (DIE). The error bars correspond to 95% confidence interval. The horizontal bars represent multiple comparisons (Bonferroni method); diamonds are the reference categories, and circles are the compare categories. Statistical significance are showed with asterisks: * *p*< 0.05, ** *p*< 0.01, *** *p*< 0.001.

Regarding ZIKV, the highly resistant strain showed higher DIR, and DIE compared with the resistant and susceptible strains, the MIR did not show differences between strains (Fig 2). The MIR presented a narrow range from 28.7% to 33.6%, and the regression model confirmed that MIR was not affected by mosquitos resistant strains (χ^2^_(2)_ = 0.75, *p* = 0.687) (Fig 2A) (S4 Table). The DIR showed marked increases with the increases in the insecticide resistance, with a wide range from 9.8% in the susceptible to 80.0% in the highly resistant strain (Fig 2B). DIR was significantly affected by the mosquito strains (χ^2^_(2)_ = 19.51, *p* = 0.0001). The DIR in the resistant strain, 41.5%, was four-fold higher, compared with the susceptible strain, 9.8%, (*z* = 3.06; *p* = 0.007), and the DIR of the highly resistant strain, 80.0%, was eight-fold higher than the susceptible strain, 9.8%, (*z* = 4.34; *p<* 0.0001) (Fig 2B). The DIR of the highly resistant strain, 80.0%, was also significantly higher, two times, compared with the resistant strain, 41.5%, (*z* = 2.41; *p =* 0.048) (S4 Table). The DIE exhibits a similar pattern to DIR, where there were proportional increases in DIE with the rise in lambda-cyhalothrin resistance. Nevertheless, this increase was less marked than the observed for DIR, ranging from 2.8% in the susceptible strain to 24.5% in highly resistant strains (Fig 2C). There was significant differences in DIE between mosquito strains (χ^2^_(2)_ = 15.97, *p* = 0.0003). The DIE of the resistant strain, 13.9%, was significantly higher, five-fold, compared with the susceptible strain, 2.8%, (*z* = 3.03; *p* = 0.007), and the DIE of the highly resistant strain, 24.5%, was even significantly higher, nine times, compared with the susceptible strain, 2.8%, (*z* = 4.00; *p<* 0.0001). However, no statistically significant differences were found between the two mosquito resistant strains (*z* = 1.64; *p =* 0.301) (Fig 2C) (S4 Table).

#### Vector competence for CHIKV

**Fig 3:**
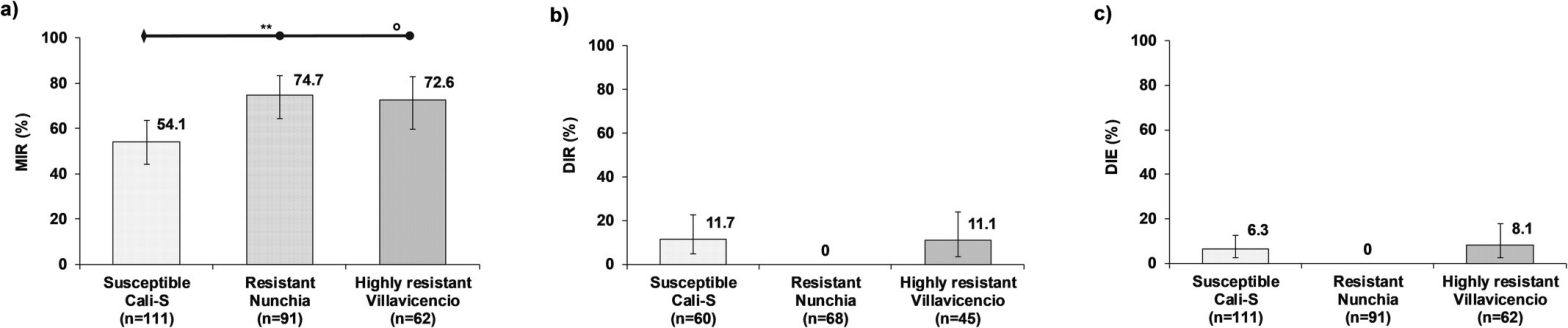
Effect of lambda-cyhalothrin resistant *Ae*. *aegypti* strains on VC for CHIKV. a) Midgut infection rate (MIR), b) Dissemination rate (DIR), and c) Dissemination efficiency (DIE). The error bars correspond to 95% confidence interval. The horizontal bars represent multiple comparisons (Bonferroni method); diamonds are the reference categories, and circles are the compare categories. Statistical significance are showed as with asterisks: * *p*< 0.05, ** *p*< 0.01, *** *p*< 0.001, ^**o**^ *p* = 0.05 (borderline significance).

For CHIKV, the two resistant strains showed higher MIR compared with the susceptible strain (Fig 3A). The regression model showed that the MIR was affected by mosquito strains (χ^2^_(2)_ = 11.01, *p* = 0.004). Multiple comparisons showed that the MIR for the resistant strain, 74.7%, was significantly higher compared with the susceptible strain, 54.1%, (*z* = 3.00; *p* = 0.008). The higher MIR of the highly resistant strain compared with the susceptible strain was on the borderline of statistical significance (*z* = 2.37; *p* = 0.054). Whereas there was no statistically significant difference between the two resistant strains (*z* = -0.30; *p* = 1.000) (Fig 3A). There was not any apparent effect of mosquito resistant strains on both DIR and DIE. Nevertheless, statistical confirmation was not possible due to the absence of CHIKV dissemination in the resistant strain and the low values of DIR and DIE in the susceptible and highly resistant strains (Fig 3B and 3C) (S5 Table).

### Genotypes and allelic frequencies of *kdr* mutations V1016I and F1534C in surviving mosquito VC bioassays

A total of 857 survivor mosquitoes of VC bioassays were genotyped for V1016I and F1534C *kdr* mutations associated with lambda-cyhalothrin resistance. For V1016I, all genotypes (wild-type, heterozygous_,_ and mutant) were present in the three *Ae*. *aegypti* strains. The I_1016_ allele_,_ associated with insecticide resistance, showed an increase in frequency, 0.08, 0.42, and 0.59 related to the increase in lambda-cyhalothrin resistance: susceptible, resistant, and highly resistant strains, respectively (Table 2). On the other hand, for the F1534C mutation, most genotypes were present in the three *Ae*. *aegypti* strains. Although the frequency of the C_1534_ allele_,_ associated with insecticide resistance, was similar and very high, 0.95–0.98, in the three *Ae*. *aegypti* strains (Table 2). Based on the Hardy-Weinberg equilibrium, the genotype frequencies of the 1016 locus of the study mosquito strains did not correspond with the Hardy-Weinberg proportion, while for the 1534 locus, only genotype frequencies of resistant and highly resistant strains corresponded with Hardy-Weinberg proportions (Table 2).

**Table 2:** Genotype and allele frequencies of the V1016I and F1534C *kdr* mutations in *Ae*. *aegypti* strains used in the vector competence bioassays and Hardy-Weinberg equilibrium test.

Locus	Mosquito resistant strains	n	Genotype frequencies			Allele frequencies		Harding Weinberg (*p*-value)
**1016**			**Wild-type**	**Heterozygous**	**Mutant**	**V**_**1016**_	**I**_**1016**_	
	Susceptible	343	0.90	0.04	0.06	0.92	0.08	<0.0001*
	Resistant	308	0.46	0.25	0.29	0.58	0.42	<0.0001*
	Highly resistant	196	0.26	0.30	0.44	0.41	0.59	<0.0001*
	**Total**	847^a^						
**1534**			**Wild-type**	**Heterozygous**	**Mutant**	**F**_**1534**_	**C**_**1534**_	
	Susceptible	350	0.01	0.04	0.95	0.03	0.97	0.0184*
	Resistant	311	0.01	0.03	0.97	0.02	0.98	0.0844
	Highly resistant	196	0.00	0.10	0.90	0.05	0.95	1.0000
	**Total**	857						

**V1016I Mutation**: V_1016_: Wild-type allele, I_1016_: Mutant allele; F1534C Mutation: F_1534_: Wild-type allele, C_1534:_ Mutant allele.

^a^ Excludes 10 mosquitos that could not be genotyped.

* Mosquito strain did not correspond with the Hardy-Weinberg proportions.

### Effect of *kdr* mutations V1016I and F1534C genotypes on vector competence variables of *Ae*. *aegypti* for DENV-2, ZIKV, and CHIKV

The following results included the effect of the genotypes of V1016I *kdr* mutations (wild-type, heterozygous and mutant) on VC for DENV-2, ZIKV, and CHIKV. The effect of F1534C *kdr* mutations is not presented because the wild-type and heterozygous genotypes were present at a very low frequency in the mosquito strains preventing any comparisons between genotypes.

### Effect of V1016I *kdr* mutation genotypes on vector competence for DENV-2

**Fig 4:**
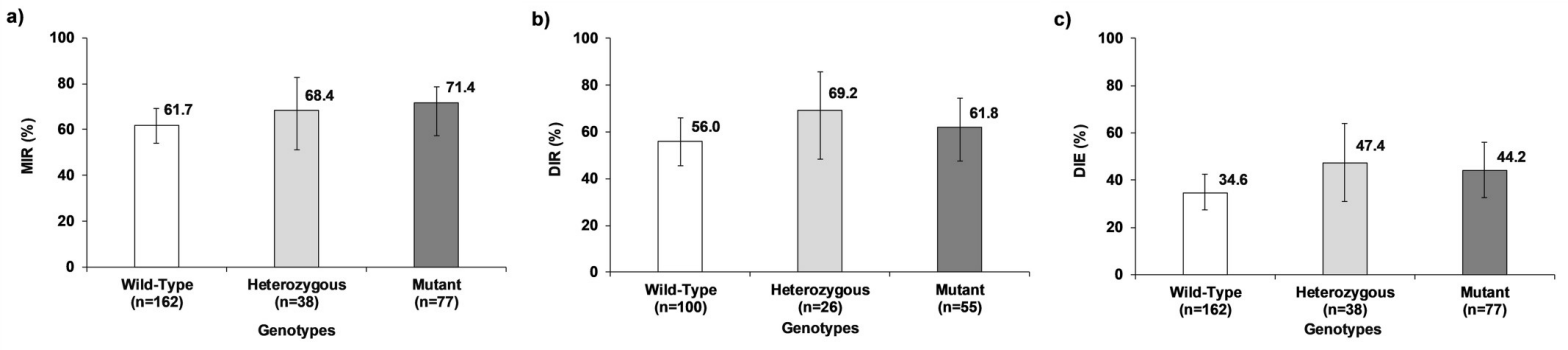
Effect of V1016I *kdr* mutation genotypes of *Ae*. *aegypti* on VC for DENV-2. a) Midgut infection rate (MIR), b) Dissemination rate (DIR), and c) Dissemination efficiency (DIE). The error bars correspond to 95% confidence interval.

For DENV-2, mutant and heterozygous genotypes of V1016I *kdr* mutation presented an apparent higher MIR, DIR, and DIE compared with the wild-type (Fig 4A–4C). The MIR ranged from 61.7% in the wild-type to 71.4% in the mutant and the regression models did not show statistically significant differences between genotypes (χ^2^_(2)_ = 2.34, *p* = 0.310). The DIR fluctuated between 56.0% in the wild-type to 69.2% in the heterozygous with no significant differences between genotypes (χ^2^_(2)_ = 1.64, *p* = 0.441). Finally, DIE ranged from 34.6% in the wild-type to 47.4% in the heterozygous, also with no significant differences between genotypes (χ^2^_(2)_ = 3.30, *p* = 0.192) (S6 Table).

### Effect of V1016I *kdr* mutation genotypes on vector competence for ZIKV

**Fig 5:**
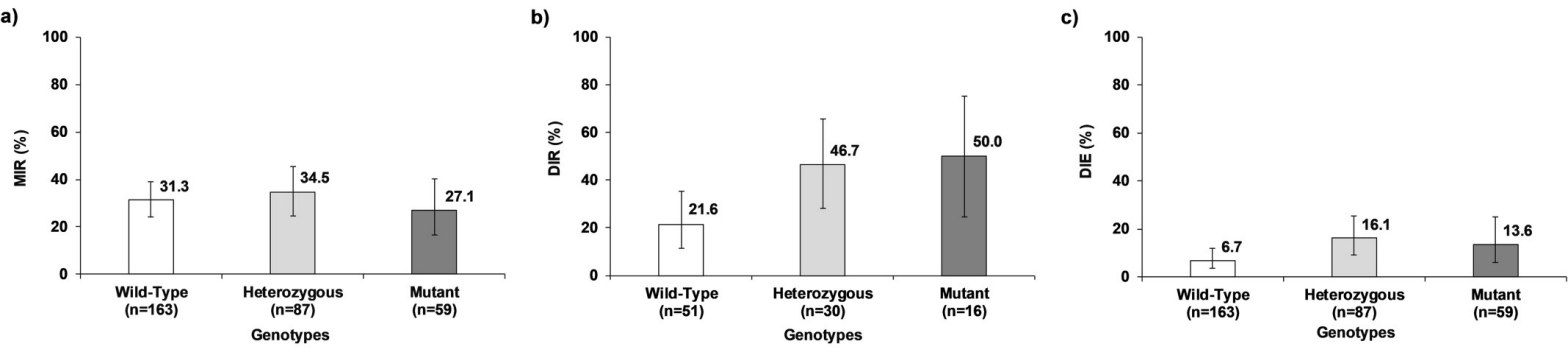
Effect of V1016I *kdr* mutation genotypes of *Ae*. *aegypti* on VC for ZIKV. a) Midgut infection rate (MIR), b) Dissemination rate (DIR), and c) Dissemination efficiency (DIE). The error bars correspond to 95% confidence interval.

Vector competence variables, except MIR, for ZIKV, presented a similar pattern described for DENV-2, where DIR and DIE for the mutant and heterozygous genotypes showed apparent higher percentages than the wild-type (Fig 5B and 5C). The DIR fluctuated between 21.6% in the wild-type to 50.0% in the mutant genotype with statistically significant differences (χ^2^_(2)_ = 7.18, *p* = 0.028) (Fig 5B). Nevertheless, multiple comparisons did not confirm significant differences (S7 Table). The heterozygous had a close to significance higher DIR, 47.5%, compared with the wild-type, 21.6% (*z* = 2.32; *p* = 0.062). The differences in DIR between mutant and wild-type and mutant and heterozygous were not significant (*z* = 2.13; *p* = 0.098; *z* = 0.22; *p* = 1.000, respectively) (S7 Table). The DIE showed also a similar pattern describe to DIR, with the highest values for the mutant, 13.6%, and the heterozygous, 16.1%, genotypes compared with the wild-type, 6.7%. Nevertheless, there was no significant effect of genotypes on DIE (χ^2^_(2)_ = 5.44, *p* = 0.063) (Fig 5C). In contrast, MIR did not present any pattern, ranging from 27.1% in the mutant to 34.5% in the heterozygous genotypes, with no significant differences between genotypes, (χ^2^_(2)_ = 0.88, *p* = 0.6427) (Fig 5A) (S7 Table).

### Effect of V1016I *kdr* mutation genotypes on vector competence for CHIKV

**Fig 6:**
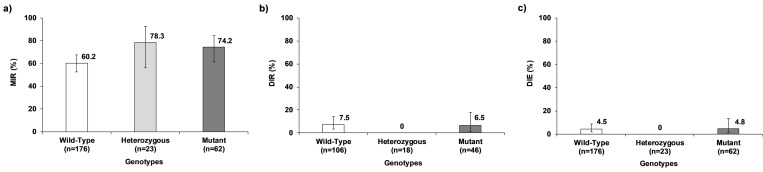
Effect of V1016I *kdr* mutation genotypes of *Ae*. *aegypti* on VC for CHIKV. a) Midgut infection rate (MIR), b) Dissemination rate (DIR), and c) Dissemination efficiency (DIE). The error bars correspond to 95% confidence interval.

The MIR for CHIKV described the same pattern shown for DENV-2 and ZIKV, with the highest MIR in the heterozygous, 78.3%, and mutant, 74.2%, genotypes compared with the wild-type, 60.2% (Fig 6A). Nevertheless, the differences in MIR were not statistically significant (χ^2^_(2)_ = 5.73, *p* = 0.057) (S8 Table). The effect of genotypes on DIR and DIE was not statistically tested due to the low values of these variables for wild-type (7.5% and 4.5%, respectively) and mutant (6.5% and 4.8%, respectively) genotypes, and the absence of DIR and DIE in the heterozygous genotype (Fig 6B and 6C) (S8 Table).

## Discussion

This study provided evidence of the effect of lambda-cyhalothrin resistance of three *Ae*. *aegypti* strains on VC variables (DIR and DIE) for ZIKV; and some evidence for DENV-2 and CHIKV. The results for ZIKV suggested for the first time a direct correlation between insecticide resistance and DIR and DIE. Furthermore, an emerging pattern of the effect of insecticide resistance on VC for ZIKV was observed: an increase in virus dissemination and no effect on infection variables.

For ZIKV infection the following general pattern of the studied VC variables was observed: for DIR and DIE, the two lambda-cyhalothrin resistant *Ae*. *aegypti* strains showed significantly higher values compared with the susceptible strains; in contrast, MIR did not present differences between resistant and susceptible strains. A similar pattern has been reported in DENV-1 for *Ae*. *aegypti* and West Nile Virus (WNV) for *Culex quinquefasciatus* [39,41]. For DENV-1 it was observed, in two *Ae*. *aegypti* strains with different levels of permethrin-resistance, that the dissemination rate (DIR and DIE, in the present study) is higher in mosquito strains with higher resistance (71%) compared with the mosquito strains with lower insecticide resistance (30,7%), and that infection rate (MIR in the present study) for DENV-1 is similar [41]. Regarding WNV, a comparison of two *Culex quinquefasciatus* strains carrying mutations (*Ester*^*2*^ and *ace-1* G119S, respectively), associated with insecticide resistance, showed that dissemination rate of the resistant strains is higher than in the susceptible strain and that there is no difference in infection rate between mutant and susceptible strains [39]. Our findings and the above mentioned literature support the emergence of a pattern on the effect of insecticide resistance on VC variables where there is an increase in dissemination rate and no effect on infection rate. This pattern suggests that insecticide resistance has a major effect on virus dissemination in mosquitos.

An apparent gradual increase in virus dissemination (DIR and DIE) with the increase in lambda-cyhalothrin resistance was observed for the ZIKV. The lowest DIR was found in the susceptible mosquito strain, 9.8%, the middle value was present in the resistant strain, 41.5%, and the highest values were found in the highly resistant mosquito strain, 80.0%. Regarding DIE, a similar trend was observed: 2.8%, 13.9%, and 25.5% for susceptible, resistant, and highly resistant strains, respectively. To our knowledge, this is the first report where a direct correlation between insecticide resistance and dissemination variables (DIR and DIE) is suggested. These results provide support for the statement, if an increase in virus dissemination is related to an increase in virus transmission, that insecticide resistance might enhance VC of *Ae*. *aegypti* for ZIKV, as has been reported for DENV-1 [41] and WNV [39], and eventually disease spread.

Taking into account that in the present study there was an apparent direct correlation between lambda-cyhalothrin resistance and DIR and DIE, we expected the observed comparative increase in I_1016_ resistance allele frequency (Susceptible: 0.08, resistant: 0.42, and highly resistant: 0.59) in *Ae*. *aegypti* resistant strains would also explain the increase in DIR and DIE for ZIKV. Nevertheless, only some evidence for DIR was found, based on the significant effect of *kdr* V1016I mutation genotypes on DIR, where there was an apparent increase in DIR for heterozygous and mutant compared with the wild-type. Currently, there is no other evidence relating mutations genotypes associated to insecticide resistance with VC in *Ae*. *aegypti*. However, this association has been described in other host-pathogens systems. In *An*. *funestus*, a study that evaluated the association between the genotypes of L119F-GSTe2 mutation and *Plasmodium* infection in pyrethroid-resistant strains showed that the homozygous resistant mosquitoes were significantly more likely to be infected with *Plasmodium* parasites compared with heterozygotes and homozygous susceptible mosquitoes [69]. Further studies will be required to confirm the effect of the V1016I *kdr* mutation on ZIKV dissemination in *Ae*. *aegypti* resistant strains and to identify other mutations associated with pyrethroid resistance that might explain changes in VC variables.

In addition, the increase in DIR and DIE for ZIKV in *Ae*. *aegypti* resistant strains also might be explained by alterations in gene expression (upregulated or downregulated) in response to infection with this virus. A study that compared transcriptomes between permethrin-resistant and permethrin-susceptible *Ae*. *aegypti* strains in response to ZIKV infection showed that there is a higher replication rate of this virus in the *Ae*. *aegypti* resistant strain and different expression levels of genes related to immunity [70], it is possible that these genes had an effect on VC in the insecticide resistant mosquito strain. Likewise, midgut microbiota may also affect insecticide resistance and VC in mosquitos. *Ae*. *aegypti* lambda-cyhalothrin resistant strains from Colombia show high mortality to lambda-cyhalothrin when previously treated with antibiotics that remove the midgut microbiota, reverting their insecticide resistant condition [37]. On the other hand, the presence of bacteria in the midgut microbiota enhances arbovirus infections in *Ae*. *aegypti* [71,72]. Finally, we could not rule out the observed differences in DIR and DIE for ZIKV between the lambda-cyhalothrin resistant *Ae*. *aegypti* strains were partially related to other intrinsic factors.

One of the intrinsic factors that modulate VC is the presence of anatomic barriers in the mosquito. There are tissue barriers including the midgut infection barrier, MIB (infection cannot be established in the midgut) and midgut escape barrier, MEB (virus replication takes place in the midgut, but dissemination to other organs does not occur) [1,73]. Regarding MEB for ZIKV, in our study, there was an apparent inverse correlation between this variable and *Ae*. *aegypti* resistant, where MEB presented a gradual decrease with the increases in insecticide resistance: 25.9% (18.9–33.9), 19.5% (13.0–27.8), and 6.1% (1.3–16.9) for susceptible, resistant and highly resistant mosquito strains, respectively, this pattern is partially supported by the no overlapping of the confidence intervals between, susceptible and highly resistant mosquito strains which represented the extreme values of insecticide resistance (S9 Table). The pattern in MEB is consistent with the direct correlations observed between the insecticide resistant and ZIKV dissemination (DIR and DIE) in the *Ae*. *aegypti* resistant strains. Concerning MIB, there were no apparent differences between the susceptible, resistant, and highly resistant strains: 71.3% (63.2–78.6), 66.4% (57.3–74.7), and 69.4% (54.6–81.7), respectively, as suggested by the overlapping confidence intervals (S9 Table). These findings for MIB and MEB support the general pattern proposed in the result that showed that insecticide resistance had an effect on virus dissemination (DIR and DIE) but not on virus infection (MIR) for ZIKV. For that, it is recommended for future studies to include dissemination variables to detect the effect of insecticide resistance on mosquito VC.

For DENV-2, the evidence for the insecticide resistance effect on VC of *Ae*. *eagypti* showed that there was a partial effect on virus dissemination, a mild increase in DIE but no effect on DIR and there was an increase in infection (MIR). For MIR the highly resistant strain showed a significantly higher MIR, 75.3%, compared with the resistant strain, 57.5%; and for DIE the highly resistant strain was higher, 50.6%, than the susceptible strain, 33.0%. Despite the differences in MIR and DIE between strains, there was no evidence for a direct correlation between insecticide resistance and VC variables as observed for ZIKV. Regarding the effect of *kdr* V1016I mutation genotypes on VC for DENV-2, regardless of the apparent higher values of MIR, DIR, and DIE of the heterozygous and mutant genotypes compared with the wild-type, the effect of the presence of this mutation on VC variables could not be statistically confirmed. Regarding the anatomic barriers, the MIB fluctuated from 24.7% (16.9–35.2) to 42.9% (32.9–53.3) in the highly resistant and resistant strains, respectively; and MEB ranged from 23.5% (15.5–33.1) to 29.9% (21.0–40.0) in resistant and susceptible strains, respectively (S9 Table). Based on the comparisons of confidence intervals, there was no apparent evidence of a relationship between these variables and the insecticide resistance of *Ae*. *aegypti*. Therefore, these findings did not support the effects of insecticide resistance on MIR and DIE, presented in the results.

Concerning CHIKV, MIR was relatively high, ranging from 54.1% to 74.7%, in the susceptible and resistant strains, respectively. In the insecticide resistance strains, the MIR had a significant increase, but this increase was not gradual to suggest a direct correlation between insecticide resistance and MIR. The finding of no differences between genotypes of *kdr* V1016I mutation related to MIR did not support the increase in MIR of the resistant *Ae*. *aegypti* strains. The high MIR for CHIKV in the resistant strains may be explained by the lowest MIB in the resistant, 25.3% (16.7–35.5) and highly resistant, 27.4% (16.9–40.2) strains compared with the susceptible strain, 45.9% (36.5–55.7). The low dissemination for CHIKV in the three *Ae*. *aegypti* strains did not allow us to detect the effect of insecticide resistance. The explanation for this low dissemination could be the high values for MEB (≥ 47%) detected for all mosquito strains (S9 Table). The high MEB may be attributed to incompatibility between CHIKV and mosquito midgut cell surface structures or to MEB dose dependence that occurred when low doses of the virus had been ingested [74]. The pattern observed for CHIKV, high infection (MIR), and very low dissemination (DIR and DIE), has been also reported for VC of *Cx*. *quinquefasciatus* for Rift valley fever virus (RVFV) where two insecticide resistant strains, with different resistant mechanisms (*Ester*^*2*^ and *ace-1* G119S), were compared with a susceptible strain [39].

In summary, the present study suggests a direct correlation between lambda-cyhalothrin resistance in *Ae*. *aegypti* and the dissemination variables (DIR and DIE) for ZIKV. This signifies that the increase in insecticide resistance leads to increases in virus dissemination on *Ae*. *aegypti*. Therefore, this finding indicates, regardless of the effect of salivary glands barriers, that the increase in insecticide resistance might enhance VC of *Ae*. *aegypti* for this virus. Future studies should include mosquito transmission variables to confirm the effect of insecticide resistance on VC of *Ae*. *aegypti* for ZIKV. For DENV-2, there was some evidence that insecticide resistance increased MIR, and DIE. Regarding CHIKV, there was only evidence that insecticide resistance increased MIR. For both DENV-2 and CHIKV future studies on infection and dissemination variables are necessary to confirm the effect of insecticide resistance on VC. Finally, in general, there was no effect of the presence of V1016I *kdr* mutations of the mosquito resistant strains on the VC variables, MIR, DIR, and DIE for the three study viruses.

## Supporting information

S1 Table Summary of viral titers of infectious blood-meals, using RT-PCR by standard curve method. (DOCX)

S2 Table Primers and thermal profile for detection of viral infection in the mosquitos and genotyping V1016I and F1534C *kdr* mutations. (DOCX)

S3 Table Effect of gradual resistant *Ae*. *aegypti* strain on MIR, DIR, and DIE for DENV-2 (Logistic regression and Bonferroni Test post-hoc pairwise). (DOCX)

S4 Table Effect of gradual resistant *Ae*. *aegypti* strain on MIR, DIR, and DIE for ZIKV (Logistic regression and Bonferroni Test post-hoc pairwise). (DOCX)

S5 Table Effect of gradual resistant *Ae*. *aegypti* strain on MIR, DIR, and DIE for CHIKV (Logistic regression and Bonferroni Test post-hoc pairwise). (DOCX)

S6 Table Effect V1016I *kdr* mutations genotypes of *Ae*. *aegypti* on MIR, DIR, and DIE for DENV-2 (Logistic regression and Bonferroni Test post-hoc pairwise). (DOCX)

S7 Table Effect V1016I *kdr* mutations genotypes of *Ae*. *aegypti* on MIR, DIR, and DIE for ZIKV (Logistic regression and Bonferroni Test post-hoc pairwise). (DOCX)

S8 Table Effect V1016I *kdr* mutations genotypes of *Ae*. *aegypti* on MIR, DIR, and DIE for CHIKV (Logistic regression and Bonferroni Test post-hoc pairwise). (DOCX)

S9 Table Data on midgut infection and head with salivary glands infection used to determine midgut infection barrier (MIB) and midgut escape barrier (MEB) percentages by *Ae*. *aegypti* strain and virus (DENV-2, ZIKV, and CHIKV). (XLSX)

S10 Table Data source for midgut, dissemination and head with salivary glands infection rates by lambda-cyhalothrin *Aedes Aegypti* resistant strain (Susceptible, resistant and, highly resistant) and *kdr* mutations: V1016I and F1534C genotype). (DOCX)
