# Supplementary material for: Vector competence of lambda-cyhalothrin resistant Aedes aegypti strains for dengue-2, Zika and chikungunya viruses in Colombia
Source: PLoS One. 2022 Oct 25;17(10):e0276493. doi: 10.1371/journal.pone.0276493 (PMC9595557; doi:10.1371/journal.pone.0276493)
Supplement: S1 Table — (DOCX) [file pone.0276493.s001.docx]

**SUPPLEMENTARY INFORMATION**

**Table S1**. Summary of viral titers of infectious blood-meals, using RT-PCR by standard curve method.

| **Number infectious blood-meals** | | **CHIKV (GCE/mL)** | **DENV-2 (GCE/mL)** | **ZIKV (GCE/mL)** |
| --- | --- | --- | --- | --- |
| 1 | | 1.81X10^7^ | 5.21 X10^6^ | 3.20 X10^10^ |
| 2 | | 3.46 X10^9^ | 3.69 X10^6^ | 1.92 X10^11^ |
| 3 | | 1.55 X10^9^ | 5.29 X10^6^ | 2.19 X10^11^ |
| 4 | | 3.46 X10^9^ | 6.01 X10^6^ | 3.17 X10^9^ |
| 5 | | 2.57 X10^7^ | 5.03 X10^6^ | 3.18 X10^10^ |
| 6 | | 1.81 X10^7^ | 4.94 X10^6^ | 1.19 X10^9^ |
| 7 | | 1.55 X10^9^ | 5.64 X10^6^ | 1.60 X10^11^ |
| 8 | | 3.46 X10^9^ | 4.05 X10^6^ | 1.24 X10^11^ |
| 9 | | 2.57 X10^7^ | 5.09 X10^6^ | 3.71 X10^10^ |
| 10 | | 1.81 X10^7^ | 5.29 X10^6^ | 9.67 X10^10^ |
| 11 | | 1.55 X10^9^ | 6.08 X10^6^ |  |
| 12 | |  | 3.91 X10^6^ |  |
| 13 | |  | 4.37 X10^6^ |  |
| 14 | |  | 4.80 X10^6^ |  |
| **Mean (GCE/mL)** | | 1.4X10^9^ | 5,0X10^6^ | 9.0X10^10^ |
| **CI 95% (GCE/mL)** | | 3.7X10^8^-2,4X10^9^ | 4.5X10^6^-5.4X10^6^ | 3.2X10^10^-1.5X10^11i^ |
| **RT-PCR**  **by standard curve method** | **LOD(GCE/ rxn)^a^** | 6.37X10^3^ | 1.56 X10^3^ | 4.68X10^1^ |
|  | **Slope** | -3.229 | -3.481 | -3.450 |
|  | **Efficiency** | 104.03% | 93,76% | 94,9% |
|  | **(r^2^)** | 0.985 | 0.983 | 0.955 |

**^a^** Limit of detection (LOD) reported by Álvarez-Díaz, D. A., Valencia-Álvarez, E., Rivera, J. A., Rengifo, A. C., Usme-Ciro, J. A., Peláez-Carvajal, D., & Lozano-Jiménez, Yenny Yolanda Torres-Fernández, O. (2021). An updated RT-qPCR assay for the simultaneous detection and quantification of chikungunya, dengue and zika viruses. Infection, Genetics and Evolution, 93, 104967. <https://doi.org/10.1016/j.meegid.2021.104967> [64].
