# Supplementary material for: Vector competence of lambda-cyhalothrin resistant Aedes aegypti strains for dengue-2, Zika and chikungunya viruses in Colombia
Source: PLoS One. 2022 Oct 25;17(10):e0276493. doi: 10.1371/journal.pone.0276493 (PMC9595557; doi:10.1371/journal.pone.0276493)
Supplement: S2 Table — (DOCX) [file pone.0276493.s002.docx]

**Table S2.** Primers and thermal profile for detection of viral infection in the mosquitos and genotyping V1016I and F1534C *kdr* mutations

| **Assays** |  | **Primers** | **Sequence (5′ → 3′)** | **Reference** | **Thermic Profile PCR** |
| --- | --- | --- | --- | --- | --- |
| *Kdr* mutations genotipification of | V1016I  Mutation | Val1016f | GCGGGCAGGGCGGCGGGGGCGGGGCCACAAATTGTTTCCCACCCGCACCGG | [66] | 95°C/10 minutes, 39 cycles: 95°C/15 seconds, 57°C/1 minute; 72°C/ 30 seconds; 72°C /2 minutes. |
|  |  | Ile1016f | GCGGGCACAAATTGTTTCCCACCCGCACTGA |  |  |
|  |  | IIe1016r | GGATGAACCSAAATTGGACAAAAGC |  |  |
|  | F1534C  Mutation | Phe1534-f | GCGGGCTCTACTTTGTGTTCTTCATCATATT | [67] | 95°C/10 minutes, 35 cycles:95°C/15 seconds, 57.5°C/1 minute; 72°C/30 seconds; melting curve:65°C to 90°C with an increase of 0.5°C/5 seconds. |
|  |  | Cys1534-f | GCGGGCAGGGCGGCGGGGGCGGGGCCTCTACTTTGTGTTCTTCATCATGTG |  |  |
|  |  | 1534r | TCTGCTCGTTGAAGTTGTCGAT |  |  |
| Detection of virus infections | Chikungunya | CHIK 6856f | TCACTCCCTGTTGGACTTGATAGA | [63] | 55°C /10 minutes, 95°C/1 minute  45 cycles: 95°C /10 seconds; 60°C/30 seconds; melting curve: 55°C to 95° C with an increase of 0.5°C/10 seconds |
|  |  | CHIK 6981 | TTGACGAACAGAGTTAGGAACATACC |  |  |
|  | Dengue-2 | D1 | TCAATATGCTGAAACGCGCGAGAAACCG | [62] | 55°C/10 minutes, 95°C /1 minute, 45 cycles: 95°C/10 seconds, 58°C/30 seconds, melting curve: 55°C to 85°C C with an increase of 0.5°C/10 seconds. |
|  |  | TS2 | CGCCACAAGGGCCATGAACAG |  |  |
|  | Zika | ZIKA-1086 | CCGCTGCCCAACACAAG | [56] | 60°C/10 minutes, 95°C/1 minute, 40 cycles: 95°C/10 seconds, 55°C/30 seconds, 72°C/30 seconds, melting curve: 72°C to 95°C C with an increase of 0.5°C/10 seconds. |
|  |  | ZIKA_1162c | CCACTAACGTTCTTTTGCAGACAT |  |  |
