# Supplementary material for: Vector competence of lambda-cyhalothrin resistant Aedes aegypti strains for dengue-2, Zika and chikungunya viruses in Colombia
Source: PLoS One. 2022 Oct 25;17(10):e0276493. doi: 10.1371/journal.pone.0276493 (PMC9595557; doi:10.1371/journal.pone.0276493)
Supplement: S3 Table — (DOCX) [file pone.0276493.s003.docx]

**Table S3.** Effect of gradual resistant *Ae. aegypti* strain on MIR, DIR, and DIE for DENV-2 (Logistic regression and Bonferroni Test post-hoc pairwise).

1. **Midgut Infection rate (MIR)**

1. 1 Model midgut infection rate (MIR) vs *Aedes aegypti* resistant strains

------------------------------------------------------------------------------------------------------------------------------------------

**Midgut infection (MIR) Odds Ratio Std. Err. z P>|z| [95% Conf. Interval]**

------------------------------------------------------------------------------------------------------------------------------------------

Susceptible strain (Cali-S)

Resistant strain (Nunchia) 0.786 0.230 -0.82 0.413 0.443 1.397

Highly resistant strain (Villavicencio) 1.798 0.589 1.79 0.073 0.946 3.419

_cons 1.694 0.356 2.51 0.012 1.122 2.558

-----------------------------------------------------------------------------------------------------------------------------------------

Note: _cons estimates baseline odds.

1.2 Model significance

----------------------------------------------------

df chi2 P>chi2

----------------------------------------------------

Strain 2 6.63 0.0364

----------------------------------------------------

Note: Bonferroni-adjusted p-values are reported for tests on individual contrasts only.

1.3 Bonferroni Test post-hoc pairwise comparison

--------------------------------------------------------------------------------------------------------------

**Bonferroni test**

**MIR** **Contrast Std. Err. z P>|z|**

--------------------------------------------------------------------------------------------------------------

**Strain**

Resistant vs Susceptible 0.239 0.293 -0.82 1.000

Highly resistant vs Susceptible 0.587 0.328 1.79 0.220

Highly resistant vs Resistant 0.827 0.324 2.55 0.032

---------------------------------------------------------------------------------------------------------------

2. **Dissemination rate (DIR)**

2.1 Model dissemination rate (DIR) vs *Aedes aegypti* resistant strains

---------------------------------------------------------------------------------------------------------------------------------------------

**Diseminacion rate (DIR) Odds Ratio Std. Err. z P>|z| [95% Conf. Interval]**

---------------------------------------------------------------------------------------------------------------------------------------------

Susceptible strain (Cali-S)

Resistant strain (Nunchia) 1.300 0.486 0.70 0.482 0.625 2.704

Highly resistant strain (Villavicencio) 1.856 0.686 1.67 0.094 0.899 3.829

_cons 1.104 0.283 0.38 0.701 0.668 1.824

-----------------------------------------------------------------------------------------------------------------------------------------

Note: _cons estimates baseline odds.

2.2 Model significance

-------------------------------------------------------

**df chi2 P>chi2**

-------------------------------------------------------

Strain 2 2.81 0.2455

--------------------------------­­­-----------------------

Note: Bonferroni-adjusted p-values are reported for tests on individual contrasts only.

2.3 Bonferroni Test post-hoc pairwise comparison

------------------------------------------------------------------------------------------------------

**Bonferroni test**

**DIR** Contrast Std. Err. z P>|z|

------------------------------------------------------------------------------------------------------

**Strain**

Resistant vs Susceptible 0.262 0.374 0.70 1.000

Highly resistant vs Susceptible 0.618 0.370 1.67 0.283

Highly resistant vs Resistant 0.356 0.380 0.94 1.000

------------------------------------------------------------------------------------------------------

**3. Dissemination efficiency (DIE)**

3.1 Dissemination efficiency (DIE) vs *Aedes aegypti* resistant strains

---------------------------------------------------------------------------------------------------------------------------------------------

**Dissemination efficiency (DIE) Odds Ratio Std. Err. z P>|z| [95% Conf. Interval]**

---------------------------------------------------------------------------------------------------------------------------------------------

Susceptible strain (Cali-S)

Resistant strain (Nunchia) 1.031 0.313 0.10 0.919 0.568 1.871

Highly resistant strain (Villavicencio) 2.080 0.636 2.39 0.017 1.141 3.789

_cons 0.492 0.106 -3.28 0.001 0.322 0.752

---------------------------------------------------------------------------------------------------------------------------------------------

Note: _cons estimates baseline odds.

3.2 Model significance

-------------------------------------------------------

**df chi2 P>chi2**

-------------------------------------------------------

Strain 2 7.33 0.0256

-------------------------------------------------------

Note: Bonferroni-adjusted p-values are reported for tests on individual contrasts only.

3.3 Bonferroni Test post-hoc pairwise comparison

----------------------------------------------------------------------------------------------------------

**Bonferroni test**

**DIE Contrast Std. Err. z P>|z|**

----------------------------------------------------------------------------------------------------------

Resistant vs Susceptible 0.031 0.304 0.10 1.000

Highly resistant vs Susceptible 0.732 0.306 2.39 0.050

Highly resistant vs Resistant 0.701 0.304 2.30 0.064

-----------------------------------------------------------------------------------------------------------
