# Supplementary material for: Vector competence of lambda-cyhalothrin resistant Aedes aegypti strains for dengue-2, Zika and chikungunya viruses in Colombia
Source: PLoS One. 2022 Oct 25;17(10):e0276493. doi: 10.1371/journal.pone.0276493 (PMC9595557; doi:10.1371/journal.pone.0276493)
Supplement: S4 Table — (DOCX) [file pone.0276493.s004.docx]

**Table S4.** Effect of gradual resistant *Ae. aegypti* strain on MIR, DIR, and DIE for ZIKV (Logistic regression and Bonferroni Test post-hoc pairwise).

1. **Midgut Infection rate (MIR)**

1. 1 Model midgut infection rate (MIR) vs *Aedes aegypti* resistant strains

------------------------------------------------------------------------------------------------------------------------------------------

**Midgut infection (MIR) Odds Ratio Std. Err. z P>|z| [95% Conf. Interval]**

------------------------------------------------------------------------------------------------------------------------------------------

Susceptible strain (Cali-S)

Resistant strain (Nunchia) 1.259 0.335 0.87 0.387 0.747 2.122

Highly resistant strain (Villavicencio) 1.097 0.396 0.26 0.796 0.541 2.227

_cons 0.402 0.074 -4.93 0.000 0.280 0.577

-----------------------------------------------------------------------------------------------------------------------------------------

Note: _cons estimates baseline odds.

1.2 Model significance

----------------------------------------------------

df chi2 P>chi2

----------------------------------------------------

Strain 2 0.75 0.6866

----------------------------------------------------

Note: Bonferroni-adjusted *p*-values are reported for tests on individual contrasts only.

1.3 Bonferroni Test post-hoc pairwise comparison

--------------------------------------------------------------------------------------------------------------

**Bonferroni test**

**MIR** **Contrast Std. Err. z P>|z|**

--------------------------------------------------------------------------------------------------------------

**Strain**

Resistant vs Susceptible 0.230 0.266 0.87 1.000

Highly resistant vs Susceptible 0.093 0.361 0.26 1.000

Highly resistant vs Resistant -0.137 0.364 -0.38 1.000

---------------------------------------------------------------------------------------------------------------

2. **Dissemination rate (DIR)**

2.1 Model dissemination rate (DIR) vs *Aedes aegypti* resistant strains

---------------------------------------------------------------------------------------------------------------------------------------------

**Diseminacion rate (DIR) Odds Ratio Std. Err. z P>|z| [95% Conf. Interval]**

---------------------------------------------------------------------------------------------------------------------------------------------

Susceptible strain (Cali-S)

Resistant strain (Nunchia) 6.552 4.026 3.06 0.002 1.965 21.846

Highly resistant strain (Villavicencio) 36.999 30.817 4.34 0.000 7.232 189.301

_cons 0.108 0.057 -4.23 0.000 0.038 0.303

---------------------------------------------------------------------------------------------------------------------------------------------

Note: _cons estimates baseline odds.

2.2 Model significance

-------------------------------------------------------

**df chi2 P>chi2**

-------------------------------------------------------

Strain 2 19.51 0.0001

--------------------------------­­­-----------------------

Note: Bonferroni-adjusted p-values are reported for tests on individual contrasts only.

2.3 Bonferroni Test post-hoc pairwise comparison

------------------------------------------------------------------------------------------------------

**Bonferroni test**

**DIR** Contrast Std. Err. z P>|z|

------------------------------------------------------------------------------------------------------

**Strain**

Resistant vs Susceptible 1.880 0.614 3.06 0.007

Highly resistant vs Susceptible 3.611 0.833 4.34 0.000

Highly resistant vs Resistant 1.731 0.719 2.41 0.048

------------------------------------------------------------------------------------------------------

**3. Dissemination efficiency (DIE)**

3.1 Dissemination efficiency (DIE) vs *Aedes aegypti* resistant strains

---------------------------------------------------------------------------------------------------------------------------------------------

**Dissemination efficiency (DIE) Odds Ratio Std. Err. z P>|z| [95% Conf. Interval]**

---------------------------------------------------------------------------------------------------------------------------------------------

Susceptible strain (Cali-S)

Resistant strain (Nunchia) 5.626 3.210 3.03 0.002 1.839 17.214

Highly resistant strain (Villavicencio) 11.270 6.833 4.00 0.000 3.435 36.981

_cons 0.029 0.015 -7.00 0.000 0.011 0.078

---------------------------------------------------------------------------------------------------------------------------------------------

Note: _cons estimates baseline odds.

3.2 Model significance

-------------------------------------------------------

**df chi2 P>chi2**

-------------------------------------------------------

Strain 2 15.97 0.0003

-------------------------------------------------------

Note: Bonferroni-adjusted p-values are reported for tests on individual contrasts only.

3.3 Bonferroni Test post-hoc pairwise comparison

----------------------------------------------------------------------------------------------------------

**Bonferroni test**

**DIE Contrast Std. Err. z P>|z|**

----------------------------------------------------------------------------------------------------------

Resistant vs Susceptible 1.727 0.570 3.03 0.007

Highly resistant vs Susceptible 2.422 0.606 4.00 0.000

Highly resistant vs Resistant 0.695 0.423 1.64 0.301

-----------------------------------------------------------------------------------------------------------
