# Supplementary material for: Vector competence of lambda-cyhalothrin resistant Aedes aegypti strains for dengue-2, Zika and chikungunya viruses in Colombia
Source: PLoS One. 2022 Oct 25;17(10):e0276493. doi: 10.1371/journal.pone.0276493 (PMC9595557; doi:10.1371/journal.pone.0276493)
Supplement: S5 Table — (DOCX) [file pone.0276493.s005.docx]

**Table S5.** Effect of gradual resistant *Ae. aegypti* strain on MIR, DIR, and DIE for CHIKV (Logistic regression and Bonferroni Test post-hoc pairwise).

1. **Midgut Infection rate (MIR)**

1. 1 Model midgut infection rate (MIR) vs *Aedes aegypti* resistant strains

------------------------------------------------------------------------------------------------------------------------------------------

**Midgut infection (MIR) Odds Ratio Std. Err. z P>|z| [95% Conf. Interval]**

------------------------------------------------------------------------------------------------------------------------------------------

Susceptible strain (Cali-S)

Resistant strain (Nunchia) 2.513 0.772 3.00 0.003 1.376 4.590

Highly resistant strain (Villavicencio) 2.25 0.771 2.37 0.018 1.150 4.403

_cons 1.176 0.224 0.85 0.393 0.810 1.709

-----------------------------------------------------------------------------------------------------------------------------------------

Note: _cons estimates baseline odds.

1.2 Model significance

----------------------------------------------------

df chi2 P>chi2

----------------------------------------------------

Strain 2 11.01 0.0041

----------------------------------------------------

Note: Bonferroni-adjusted *p*-values are reported for tests on individual contrasts only.

1.3 Bonferroni Test post-hoc pairwise comparison

--------------------------------------------------------------------------------------------------------------

**Bonferroni test**

**MIR** **Contrast Std. Err. z P>|z|**

--------------------------------------------------------------------------------------------------------------

**Strain**

Resistant vs Susceptible 0.921 0.307 3.00 0.008

Highly resistant vs Susceptible 0.811 0.342 2.37 0.054

Highly resistant vs Resistant -0.110 0.373 -0.30 1.000

---------------------------------------------------------------------------------------------------------------

2. **Dissemination rate (DIR)**

2.1 Model dissemination rate (DIR) vs *Aedes aegypti* resistant strains

---------------------------------------------------------------------------------------------------------------------------------------------

**Diseminacion rate (DIR) Odds Ratio Std. Err. z P>|z| [95% Conf. Interval]**

---------------------------------------------------------------------------------------------------------------------------------------------

Susceptible strain (Cali-S)

Resistant strain (Nunchia) 1 (empty)

Highly resistant strain (Villavicencio) 0.946 0.589 -0.09 0.929 0.280 3.202

_cons 0.132 0.053 -5.03 0.000 0.060 0.290

---------------------------------------------------------------------------------------------------------------------------------------------

Note: _cons estimates baseline odds.

2.2 Model significance

-------------------------------------------------------

**df chi2 P>chi2**

-------------------------------------------------------

Strain (not testable)

-------------------------------------------------------

Note: Bonferroni-adjusted *p*-values are reported for tests on individual contrasts only.

2.3 Bonferroni Test post-hoc pairwise comparison

------------------------------------------------------------------------------------------------------

**Bonferroni test**

**DIR** Contrast Std. Err. z P>|z|

------------------------------------------------------------------------------------------------------

**Strain**

Resistant vs Susceptible (not estimable)

Highly resistant vs Susceptible -0.055 0.622 -0.09 1.000

Highly resistant vs Resistant (not estimable)

------------------------------------------------------------------------------------------------------

**3. Dissemination efficiency (DIE)**

3.1 Dissemination efficiency (DIE) vs *Aedes aegypti* resistant strains

---------------------------------------------------------------------------------------------------------------------------------------------

**Dissemination efficiency (DIE) Odds Ratio Std. Err. z P>|z| [95% Conf. Interval]**

---------------------------------------------------------------------------------------------------------------------------------------------

Susceptible strain (Cali-S)

Resistant strain (Nunchia) 1 (empty)

Highly resistant strain (Villavicencio) 1.303 0.793 0.44 0.663 0.395 4.293

_cons 0.067 0.026 -6.91 0.000 0.031 0.145

---------------------------------------------------------------------------------------------------------------------------------------------

Note: _cons estimates baseline odds.

3.2 Model significance

-------------------------------------------------------

**df chi2 P>chi2**

-------------------------------------------------------

Strain (not testable)

-------------------------------------------------------

Note: Bonferroni-adjusted *p*-values are reported for tests on individual contrasts only.

3.3 Bonferroni Test post-hoc pairwise comparison

----------------------------------------------------------------------------------------------------------

**Bonferroni test**

**DIE Contrast Std. Err. z P>|z|**

----------------------------------------------------------------------------------------------------------

Resistant vs Susceptible (not estimable)

Highly resistant vs Susceptible 0.265 0.608 0.44 1.000

Highly resistant vs Resistant (not estimable)

-----------------------------------------------------------------------------------------------------------
