# Supplementary material for: Vector competence of lambda-cyhalothrin resistant Aedes aegypti strains for dengue-2, Zika and chikungunya viruses in Colombia
Source: PLoS One. 2022 Oct 25;17(10):e0276493. doi: 10.1371/journal.pone.0276493 (PMC9595557; doi:10.1371/journal.pone.0276493)
Supplement: S6 Table — (DOCX) [file pone.0276493.s006.docx]

**Table S6.** Effect V1016I *kdr* mutations genotypes of *Ae. aegypti* on MIR, DIR, and DIE for DENV-2 (Logistic regression and Bonferroni Test post-hoc pairwise).

1. **Midgut Infection rate (MIR)**

1. 1 Model midgut infection rate (MIR) vs V1016I *kdr* mutations genotypes of *Ae. aegypti*

------------------------------------------------------------------------------------------------------------------------------------------

**Midgut infection (MIR) Odds Ratio Std. Err. z P>|z| [95% Conf. Interval]**

------------------------------------------------------------------------------------------------------------------------------------------

Wild-type

Heterozygous 1.343 0.517 0.77 0.443 0.632 - 2.855

Mutant 1.550 0.464 1.46 0.144 0.861 - 2.788

_cons 1.613 0.261 2.96 0.003 1.175 - 2.214

-----------------------------------------------------------------------------------------------------------------------------------------

Note: _cons estimates baseline odds.

1.2 Model significance

----------------------------------------------------

df chi2 P>chi2

----------------------------------------------------

Genotype 2 2.34 0.3104

----------------------------------------------------

Note: Bonferroni-adjusted p-values are reported for tests on individual contrasts only.

1.3 Bonferroni Test post-hoc pairwise comparison

--------------------------------------------------------------------------------------------------------------

**Bonferroni test**

**MIR** **Contrast Std. Err. z P>|z|**

--------------------------------------------------------------------------------------------------------------

**Genotype**

Heterozygous vs Wild-type 0.295 0.385 0.77 1.000

Mutant vs Wild-type 0.438 0.299 1.46 0.431

Mutant vs Heterozygous 0.143 0.430 0.33 1.000

---------------------------------------------------------------------------------------------------------------

2. **Dissemination rate (DIR)**

2.1 Model dissemination rate (DIR) vs V1016I *kdr* mutations genotypes of *Ae. aegypti*

---------------------------------------------------------------------------------------------------------------------------------------------

**Diseminacion rate (DIR) Odds Ratio Std. Err. z P>|z| [95% Conf. Interval]**

---------------------------------------------------------------------------------------------------------------------------------------------

Wild-type

Heterozygous 1.768 0.831 1.21 0.226 0.703 - 4.443

Mutant 1.272 0.436 0.70 0.483 0.649 - 2.491

_cons 1.273 0.256 1.20 0.231 0.857 - 1.889

---------------------------------------------------------------------------------------------------------------------------------------------

Note: _cons estimates baseline odds.

2.2 Model significance

-------------------------------------------------------

**df chi2 P>chi2**

-------------------------------------------------------

Genotype 2 1.64 0.4413

-------------------------------------------------------

Note: Bonferroni-adjusted p-values are reported for tests on individual contrasts only.

2.3 Bonferroni Test post-hoc pairwise comparison

------------------------------------------------------------------------------------------------------

**Bonferroni test**

**DIR** Contrast Std. Err. z P>|z|

------------------------------------------------------------------------------------------------------

**Genotype**

Heterozygous vs Wild-type 0.570 0.470 1.21 0.677

Mutant vs Wild-type 0.241 0.343 0.70 1.000

Mutant vs Heterozygous -0.329 0.507 -0.65 1.000

------------------------------------------------------------------------------------------------------

**3. Dissemination efficiency (DIE)**

3.1 Dissemination efficiency (DIE) vs V1016I *kdr* mutations genotypes of *Ae. aegypti*

---------------------------------------------------------------------------------------------------------------------------------------------

**Dissemination efficiency (DIE) Odds Ratio Std. Err. z P>|z| [95% Conf. Interval]**

---------------------------------------------------------------------------------------------------------------------------------------------

Wild-type

Heterozygous 1.703 0.621 1.46 0.144 0.834 3.480

Mutant 1.497 0.423 1.43 0.154 0.860 2.605

_cons 0.528 0.087 -3.86 0.000 0.382 0.730

---------------------------------------------------------------------------------------------------------------------------------------------

Note: _cons estimates baseline odds.

3.2 Model significance

-------------------------------------------------------

**df chi2 P>chi2**

-------------------------------------------------------

Genotype 2 3.30 0.1923

-------------------------------------------------------

Note: Bonferroni-adjusted p-values are reported for tests on individual contrasts only.

3.3 Bonferroni Test post-hoc pairwise comparison

----------------------------------------------------------------------------------------------------------

**Bonferroni test**

**DIE Contrast Std. Err. z P>|z|**

----------------------------------------------------------------------------------------------------------

**Genotype**

Heterozygous vs Wild-type 0.533 0.364 1.46 0.432

Mutant vs Wild-type 0.403 0.283 1.43 0.462

Mutant vs Heterozygous -0.129 0.398 -0.33 1.000

-----------------------------------------------------------------------------------------------------------
