# Supplementary material for: Vector competence of lambda-cyhalothrin resistant Aedes aegypti strains for dengue-2, Zika and chikungunya viruses in Colombia
Source: PLoS One. 2022 Oct 25;17(10):e0276493. doi: 10.1371/journal.pone.0276493 (PMC9595557; doi:10.1371/journal.pone.0276493)
Supplement: S7 Table — (DOCX) [file pone.0276493.s007.docx]

**Table S7.** Effect V1016I *kdr* mutations genotypes of *Ae. aegypti* on MIR, DIR, and DIE for ZIKV (Logistic regression and Bonferroni Test post-hoc pairwise).

1. **Midgut Infection rate (MIR)**

1. 1 Model midgut infection rate (MIR) vs V1016I *kdr* mutations genotypes of *Ae. aegypti*

------------------------------------------------------------------------------------------------------------------------------------------

**Midgut infection (MIR) Odds Ratio Std. Err. z P>|z| [95% Conf. Interval]**

------------------------------------------------------------------------------------------------------------------------------------------

Wild-type

Heterozygous 1.156 0.326 0.51 0.607 0.665 2.008

Mutant 0.817 0.276 -0.60 0.550 0.421 1.585

_cons 0.455 0.077 -4.66 0.000 0.327 0.634

-----------------------------------------------------------------------------------------------------------------------------------------

Note: _cons estimates baseline odds.

1.2 Model significance

----------------------------------------------------

df chi2 P>chi2

----------------------------------------------------

Genotype 2 0.88 0.6427

----------------------------------------------------

Note: Bonferroni-adjusted *p*-values are reported for tests on individual contrasts only.

1.3 Bonferroni Test post-hoc pairwise comparison

--------------------------------------------------------------------------------------------------------------

**Bonferroni test**

**MIR** **Contrast Std. Err. z P>|z|**

--------------------------------------------------------------------------------------------------------------

**Genotype**

Heterozygous vs Wild-type 0.145 0.282 0.51 1.000

Mutant vs Wild-type -0.202 0.338 -0.60 1.000

Mutant vs Heterozygous -0.347 0.370 -0.94 1.000

---------------------------------------------------------------------------------------------------------------

2. **Dissemination rate (DIR)**

2.1 Model dissemination rate (DIR) vs V1016I *kdr* mutations genotypes of *Ae. aegypti*

---------------------------------------------------------------------------------------------------------------------------------------------

**Diseminacion rate (DIR) Odds Ratio Std. Err. z P>|z| [95% Conf. Interval]**

---------------------------------------------------------------------------------------------------------------------------------------------

Wild-type

Heterozygous 3.182 1.590 2.32 0.021 1.194 8.475

Mutant 3.636 2.200 2.13 0.033 1.111 11.900

_cons 0.275 0.094 -3.79 0.000 0.141 0.536

---------------------------------------------------------------------------------------------------------------------------------------------

Note: _cons estimates baseline odds.

2.2 Model significance

-------------------------------------------------------

**df chi2 P>chi2**

-------------------------------------------------------

Genotype 2 7.18 0.0275

-------------------------------------------------------

Note: Bonferroni-adjusted *p*-values are reported for tests on individual contrasts only.

2.3 Bonferroni Test post-hoc pairwise comparison

------------------------------------------------------------------------------------------------------

**Bonferroni test**

**DIR** Contrast Std. Err. z P>|z|

------------------------------------------------------------------------------------------------------

**Genotype**

Heterozygous vs Wild-type 1.157 0.500 2.32 0.062

Mutant vs Wild-type 1.291 0.605 2.13 0.098

Mutant vs Heterozygous 0.133 0.620 0.22 1.000

------------------------------------------------------------------------------------------------------

**3. Dissemination efficiency (DIE)**

3.1 Dissemination efficiency (DIE) vs V1016I *kdr* mutations genotypes of *Ae. aegypti*

---------------------------------------------------------------------------------------------------------------------------------------------

**Dissemination efficiency (DIE) Odds Ratio Std. Err. z P>|z| [95% Conf. Interval]**

---------------------------------------------------------------------------------------------------------------------------------------------

Wild-type

Heterozygous 2.650 1.132 2.28 0.023 1.147 6.123

Mutant 2.167 1.066 1.57 0.116 0.826 5.686

_cons 0.072 0.022 -8.41 0.000 0.039 0.133

---------------------------------------------------------------------------------------------------------------------------------------------

Note: _cons estimates baseline odds.

3.2 Model significance

-------------------------------------------------------

**df chi2 P>chi2**

-------------------------------------------------------

Genotype 2 5.54 0.0627

-------------------------------------------------------

Note: Bonferroni-adjusted *p*-values are reported for tests on individual contrasts only.

3.3 Bonferroni Test post-hoc pairwise comparison

----------------------------------------------------------------------------------------------------------

**Bonferroni test**

**DIE Contrast Std. Err. z P>|z|**

----------------------------------------------------------------------------------------------------------

**Genotype**

Heterozygous vs Wild-type 0.974 0.427 2.28 0.068

Mutant vs Wild-type 0.774 0.492 1.57 0.348

Mutant vs Heterozygous -0.201 0.479 -0.42 1.000

-----------------------------------------------------------------------------------------------------------
