# Supplementary material for: Vector competence of lambda-cyhalothrin resistant Aedes aegypti strains for dengue-2, Zika and chikungunya viruses in Colombia
Source: PLoS One. 2022 Oct 25;17(10):e0276493. doi: 10.1371/journal.pone.0276493 (PMC9595557; doi:10.1371/journal.pone.0276493)
Supplement: S8 Table — (DOCX) [file pone.0276493.s008.docx]

**Table S8.** Effect V1016I *kdr* mutations genotypes of *Ae. aegypti* on MIR, DIR, and DIE for CHIKV (Logistic regression and Bonferroni Test post-hoc pairwise).

1. **Midgut Infection rate (MIR)**

1. 1 Model midgut infection rate (MIR) vs V1016I *kdr* mutations genotypes of *Ae. aegypti*

------------------------------------------------------------------------------------------------------------------------------------------

**Midgut infection (MIR) Odds Ratio Std. Err. z P>|z| [95% Conf. Interval]**

------------------------------------------------------------------------------------------------------------------------------------------

Wild-type

Heterozygous 2.377 1.256 1.64 0.101 0.844 6.698

Mutant 1.898 0.624 1.95 0.051 0.997 3.615

_cons 1.514 0.233 2.69 0.007 1.120 2.048

-----------------------------------------------------------------------------------------------------------------------------------------

Note: _cons estimates baseline odds.

1.2 Model significance

----------------------------------------------------

df chi2 P>chi2

----------------------------------------------------

Genotype 2 5.73 0.0571

----------------------------------------------------

Note: Bonferroni-adjusted *p*-values are reported for tests on individual contrasts only.

1.3 Bonferroni Test post-hoc pairwise comparison

--------------------------------------------------------------------------------------------------------------

**Bonferroni test**

**MIR** **Contrast Std. Err. z P>|z|**

--------------------------------------------------------------------------------------------------------------

**Genotype**

Heterozygous vs Wild-type 0.866 0.528 1.64 0.304

Mutant vs Wild-type 0.641 0.328 1.95 0.153

Mutant vs Heterozygous -0.225 0.583 -0.39 1.000

---------------------------------------------------------------------------------------------------------------

2. **Dissemination rate (DIR)**

2.1 Model dissemination rate (DIR) vs V1016I *kdr* mutations genotypes of *Ae. aegypti*

---------------------------------------------------------------------------------------------------------------------------------------------

**Diseminacion rate (DIR) Odds Ratio Std. Err. z P>|z| [95% Conf. Interval]**

---------------------------------------------------------------------------------------------------------------------------------------------

Wild-type

Heterozygous 1 (empty)

Mutant 0.855 0.599 -0.22 0.823 0.216 3.378

_cons 0.082 0.030 -6.81 0.000 0.040 0.168

---------------------------------------------------------------------------------------------------------------------------------------------

Note: _cons estimates baseline odds.

2.2 Model significance

-------------------------------------------------------

**df chi2 P>chi2**

-------------------------------------------------------

Genotype (not testable)

-------------------------------------------------------

Note: Bonferroni-adjusted *p*-values are reported for tests on individual contrasts only.

2.3 Bonferroni Test post-hoc pairwise comparison

------------------------------------------------------------------------------------------------------

**Bonferroni test**

**DIR** Contrast Std. Err. z P>|z|

------------------------------------------------------------------------------------------------------

**Genotype**

Heterozygous vs Wild-type (not estimable)

Mutant vs Wild-type -0.157 0.701 -0.22 1.000

Mutant vs Heterozygous (not estimable)

------------------------------------------------------------------------------------------------------

**3. Dissemination efficiency (DIE)**

3.1 Dissemination efficiency (DIE) vs V1016I *kdr* mutations genotypes of *Ae. aegypti*

---------------------------------------------------------------------------------------------------------------------------------------------

**Dissemination efficiency (DIE) Odds Ratio Std. Err. z P>|z| [95% Conf. Interval]**

---------------------------------------------------------------------------------------------------------------------------------------------

Wild-type

Heterozygous 1(empty)

Mutant 1.068 0.741 0.09 0.925 0.274 4.159

_cons 0.048 0.017 -8.41 0.000 0.023 0.097

---------------------------------------------------------------------------------------------------------------------------------------------

Note: _cons estimates baseline odds.

3.2 Model significance

-------------------------------------------------------

**df chi2 P>chi2**

-------------------------------------------------------

Genotype (not testable)

-------------------------------------------------------

Note: Bonferroni-adjusted *p*-values are reported for tests on individual contrasts only.

3.3 Bonferroni Test post-hoc pairwise comparison

----------------------------------------------------------------------------------------------------------

**Bonferroni test**

**DIE Contrast Std. Err. z P>|z|**

----------------------------------------------------------------------------------------------------------

**Genotype**

Heterozygous vs Wild-type (not estimable)

Mutant vs Wild-type 0.065 0.694 0.09 1.000

Mutant vs Heterozygous (not estimable)

-----------------------------------------------------------------------------------------------------------
