# Supplementary material for: Vector competence of lambda-cyhalothrin resistant Aedes aegypti strains for dengue-2, Zika and chikungunya viruses in Colombia
Source: PLoS One. 2022 Oct 25;17(10):e0276493. doi: 10.1371/journal.pone.0276493 (PMC9595557; doi:10.1371/journal.pone.0276493)
Supplement: S10 Table — (DOCX) [file pone.0276493.s010.docx]

**Table S10.** Data source for midgut, dissemination and head with salivary glands infection rates by lambda-cyhalothrin *Aedes Aegypti* resistant strain (Susceptible, resistant and, highly resistant) and *kdr* mutations: V1016I and F1534C genotype).

| Infection | Mutation | Genotype *kdr* mutation | Variable | Chikungunya virus | | | Dengue-2 virus | | | Zika virus | | |
| --- | --- | --- | --- | --- | --- | --- | --- | --- | --- | --- | --- | --- |
|  |  |  |  | **Susceptible (Cali-S)** | **Resistant**  **Nunchia** | **Highly resistant**  **Villavicencio** | **Susceptible (Cali-S)** | **Resistant**  **Nunchia** | **Highly resistant**  **Villavicencio** | **Susceptible (Cali-S)** | **Resistant**  **Nunchia** | **Highly resistant**  **Villavicencio** |
| Midgut infection  (MIR) | Total |  | Survivor mosquitoes | 111 | 91 | 62 | 97 | 98 | 85 | 143 | 122 | 49 |
|  |  |  | PCR-Positive | 60 | 68 | 45 | 61 | 56 | 64 | 41 | 41 | 15 |
|  |  |  | Midgut infection rate(%) | 54.05 | 74.73 | 72.58 | 62.89 | 57.14 | 75.29 | 28.67 | 33.61 | 30.61 |
|  |  |  | CI95% (%) | 44.33-63.55 | 64.53-83.25 | 59.77-83.15 | 52.48- 72.48 | 46.75 - 67.1 | 64.75-84.01 | 21.42-36.82 | 25.3-42.72 | 18.25 - 45.41 |
|  | V1016I | Wild-type | Survivor mosquitoes | 103 | 52 | 21 | 94 | 48 | 20 | 112 | 41 | 10 |
|  |  |  | PCR-Positive | 55 | 34 | 17 | 59 | 23 | 18 | 35 | 13 | 3 |
|  |  |  | Midgut infection rate(%) | 53.4 | 65.38 | 80.95 | 62.77 | 47.92 | 90 | 31.25 | 31.71 | 30 |
|  |  |  | CI95% (%) | 43.30-63.29 | 50.91-78.03 | 58.09-94.55 | 52.28-72.52 | 33.29-62.81 | 68.30-98.77 | 22.83-40.70 | 18.08-48.09 | 6.67-65.25 |
|  |  | Heterozygous | Survivor mosquitoes | 4 | 9 | 10 | 1 | 17 | 20 | 8 | 51 | 28 |
|  |  |  | PCR-Positive | 2 | 8 | 8 | 1 | 11 | 14 | 2 | 19 | 9 |
|  |  |  | Midgut infection rate(%) | 50 | 88.89 | 80 | 100 | 64.71 | 70 | 25 | 37.25 | 32.14 |
|  |  |  | CI95% (%) | 6.76-93.24 | 51.75-99.72 | 44.39-97.48 | 2.5-1* | 38.33-85.79 | 45.72-88.11 | 3.19-65.09 | 24.13-51.92 | 15.88-52.35 |
|  |  | Mutant | Survivor mosquitoes | 1 | 30 | 31 | 2 | 30 | 45 | 18 | 30 | 11 |
|  |  |  | PCR-Positive | 0 | 26 | 20 | 1 | 22 | 32 | 4 | 9 | 3 |
|  |  |  | Midgut infection rate(%) | 0 | 86.67 | 64.52 | 50 | 73.33 | 71.11 | 22.22 | 30 | 27.27 |
|  |  |  | CI95% (%) | 0-97.5* | 69.28-96.24 | 45.37-80.77 | 1.26-98.74 | 54.11-87.72 | 55.69-83.63 | 6.41-47.64 | 14.73-49.40 | 6.02-60.97 |
|  | P1534C | Wild-type | Survivor mosquitoes | 0 | 0 | 0 | 0 | 1 | 0 | 2 | 0 | 0 |
|  |  |  | PCR-Positive |  |  |  |  | 0 |  | 0 |  |  |
|  |  |  | Midgut infection rate(%) |  |  |  |  | 0 |  | 100 |  |  |
|  |  |  | CI95% (%) |  |  |  |  | 0-97.5* |  | 0-84.19* |  |  |
|  |  | Heterozygous | Survivor mosquitoes | 4 | 2 | 7 | 0 | 7 | 6 | 10 | 0 | 7 |
|  |  |  | PCR-Positive | 0 | 2 | 6 |  | 6 | 5 | 1 |  | 2 |
|  |  |  | Midgut infection rate(%) | 0 | 100 | 85.71 |  | 85.71 | 83.33 | 10.00 |  | 28.57 |
|  |  |  | CI95% (%) | 0-60.24* | 15.81-1* | 42.13-99.64 |  | 42.13-99.64 | 35.88-99.58 | 0.25-44.5 |  | 3.67-70.96 |
|  |  | Mutant | Survivor mosquitoes | 106 | 89 | 54 | 97 | 90 | 79 | 131 | 122 | 42 |
|  |  |  | PCR-Positive | 59 | 66 | 38 | 61 | 50 | 59 | 40 | 41 | 13 |
|  |  |  | Midgut infection rate(%) | 55.66 | 74.16 | 70.37 | 62.89 | 55.56 | 74.68 | 30.53 | 33.61 | 30.95 |
|  |  |  | CI95% (%) | 45.69-65.31 | 63.79-82.86 | 56.39-82.02 | 52.48-72.48 | 44.70-66.04 | 63.64-83.80 | 22.79-39.18 | 25.31-42.72 | 17.62-47.09 |
| Head with salivary glands infection  (DIE) | Total |  | Survivor mosquitoes | 111 | 91 | 62 | 97 | 98 | 85 | 143 | 122 | 49 |
|  |  |  | PCR-positive | 7 | 0 | 5 | 32 | 33 | 43 | 4 | 17 | 12 |
|  |  |  | Salivary glands infection rate (%) | 6.31 | 0 | 8.06 | 32.99 | 33.67 | 50.59 | 2.8 | 13.93 | 24.49 |
|  |  |  | CI95% (%) | 2.57-12.56 | 0-3.97* | 2.67-17.83 | 23.78-43.27 | 24.44-43.93 | 39.52-61.61 | 0.77-7.00 | 8.33-21.37 | 13.34-38.87 |
|  | V1016I | Wild-type | Survivor mosquitoes | 103 | 52 | 21 | 94 | 48 | 20 | 112 | 41 | 10 |
|  |  |  | PCR-positive | 6 | 0 | 2 | 30 | 17 | 9 | 3 | 5 | 3 |
|  |  |  | Salivary glands infection rate (%) | 5.83 | 0 | 9.52 | 31.91 | 35.42 | 45.00 | 2.68 | 12.20 | 30.00 |
|  |  |  | CI95% (%) | 2.17-12.25 | 0-6.84* | 1.17-30.38 | 22.67-42.33 | 22.16-50.54 | 23.06-68.47 | 0.06-7.63 | 4.08-26.20 | 6.67- 65.25 |
|  |  | Heterozygous | Survivor mosquitoes | 4 | 9 | 10 | 1 | 17 | 20 | 8 | 51 | 28 |
|  |  |  | PCR-positive | 0 | 0 | 0 | 1 | 6 | 11 | 0 | 7 | 7 |
|  |  |  | Salivary glands infection rate (%) | 0 | 0 | 0 | 100 | 35.29 | 55.00 | 0 | 13.73 | 25.00 |
|  |  |  | CI95% (%) | 0-60.24* | 0-33.63* | 0-30.85* | 2.5-1* | 14.21-61.67 | 31.53-76.94 | 0-36.94* | 5.70-26.26 | 10.69-44.87 |
|  |  | Mutant | Survivor mosquitoes | 1 | 30 | 31 | 2 | 30 | 45 | 18 | 30 | 11 |
|  |  |  | PCR-positive | 0 | 0 | 3 | 1 | 10 | 23 | 1 | 5 | 2 |
|  |  |  | Salivary glands infection rate (%) | 0 | 0 | 9.68 | 50 | 33.33 | 51.11 | 5.56 | 16.67 | 18.18 |
|  |  |  | CI95% (%) | 0-97.5* | 0-11.57* | 2.04-25.75 | 1.26-98.74 | 17.29-52.81 | 35.77-66.30 | 0.14-27.29 | 5.64-34.72 | 2.28-51.78 |
|  | P1534C | Wild-type | Survivor mosquitoes | 0 | 0 | 0 | 0 | 1 | 0 | 2 | 0 | 0 |
|  |  |  | PCR-positive |  |  |  |  | 0 |  | 0 |  |  |
|  |  |  | Salivary glands infection rate (%) |  |  |  |  | 0 |  | 100 |  |  |
|  |  |  | CI95% (%) |  |  |  |  | 0-97.5* |  | 0-84.19* |  |  |
|  |  | Heterozygous | Survivor mosquitoes | 4 | 2 | 7 | 0 | 7 | 6 | 10 | 0 | 7 |
|  |  |  | PCR-positive | 0 | 0 | 0 |  | 2 | 3 | 0 |  | 1 |
|  |  |  | Salivary glands infection rate (%) | 0 | 0 | 0 |  | 28.57 | 50.00 | 0 |  | 14.29 |
|  |  |  | CI95% (%) | 0-60.24* | 0-84.19* | 0-40.96* |  | 3.67-70.96 | 11.81-88.19 | 0-30.85* |  | 0.36-57.87 |
|  |  | Mutant | Survivor mosquitoes | 106 | 89 | 54 | 97 | 90 | 79 | 131 | 122 | 42 |
|  |  |  | PCR-positive | 7 | 0 | 5 | 32 | 31 | 40 | 4 | 17 | 11 |
|  |  |  | Salivary glands infection rate (%) | 6.60 | 0 | 9.26 | 32.99 | 34.44 | 50.63 | 3.05 | 13.93 | 26.19 |
|  |  |  | CI95% (%) | 2.70-13.13 | 0-4.06* | 3.08-20.30 | 23.78-43.27 | 24.74-45.20 | 39.14-62.08 | 0.84-7.63 | 8.33-21.37 | 13.86-42.04 |

| Dissemination  (DIR) | Total |  | Survivor mosquitoes | 60 | 68 | 45 | 61 | 56 | 64 | 41 | 41 | 15 |
| --- | --- | --- | --- | --- | --- | --- | --- | --- | --- | --- | --- | --- |
|  |  |  | PCR-positive | 7 | 0 | 5 | 32 | 33 | 43 | 4 | 17 | 12 |
|  |  |  | Dissemination rate (%) | 11.67 | 0 | 11.11 | 52.46 | 58.93 | 67.19 | 9.76 | 41.46 | 80 |
|  |  |  | CI95% (%) | 4.82-22.57 | 0-5.28* | 3.71- 24.05 | 39.27-65.40 | 44.98-71.90 | 54.31- 78.41 | 2.72- 23.13 | 26.32- 57.89 | 51.91- 95.67 |
|  | V1016I | Wild-type | Survivor mosquitoes | 55 | 34 | 17 | 59 | 23 | 18 | 35 | 13 | 3 |
|  |  |  | PCR-positive | 6 | 0 | 2 | 30 | 17 | 9 | 3 | 5 | 3 |
|  |  |  | Dissemination rate (%) | 10.91 | 0 | 11.76 | 50.85 | 73.91 | 50 | 8.57 | 38.46 | 100 |
|  |  |  | CI95% (%) | 4.11-22.25 | 0-10.28* | 1.46-36.44 | 37.50-64.11 | 51.59-89.77 | 26.02-73.98 | 1.80-23.06 | 13.86-68.42 | 29.24-1* |
|  |  | Heterozygous | Survivor mosquitoes | 2 | 8 | 8 | 1 | 11 | 14 | 2 | 19 | 9 |
|  |  |  | PCR-positive | 0 | 0 | 0 | 1 | 6 | 11 | 0 | 7 | 7 |
|  |  |  | Dissemination rate (%) | 0 | 0 | 0 | 100 | 54.55 | 78.57 | 0 | 36.84 | 77.78 |
|  |  |  | CI95% (%) | 0-84.19* | 0-36.94* | 0-36.94* | 2.5-1* | 23.38-83.25 | 49.20-95.34 | 0-84.19* | 16.29-61.64 | 39.99-97.19 |
|  |  | Mutant | Survivor mosquitoes | 0 | 26 | 20 | 1 | 22 | 32 | 4 | 9 | 3 |
|  |  |  | PCR-positive |  | 0 | 3 | 1 | 10 | 23 | 1 | 5 | 2 |
|  |  |  | Dissemination rate (%) |  | 0 | 15 | 100 | 45.45 | 71.88 | 25 | 55.56 | 66.67 |
|  |  |  | CI95% (%) |  | 0-13.23* | 3.21-37.89 | 2.5-1* | 24.39-67.79 | 53.25-86.25 | 0.63-80.59 | 21.20-86.30 | 9.43-99.16 |
|  | P1534C | Wild-type | Survivor mosquitoes | 0 | 0 | 0 | 0 | 0 | 0 | 0 | 0 | 0 |
|  |  |  | PCR-positive |  |  |  |  |  |  |  |  |  |
|  |  |  | Dissemination rate (%) |  |  |  |  |  |  |  |  |  |
|  |  |  | CI95% (%) |  |  |  |  |  |  |  |  |  |
|  |  | Heterozygous | Survivor mosquitoes | 0 | 2 | 6 | 0 | 6 | 5 | 1 | 0 | 2 |
|  |  |  | PCR-positive |  | 2 | 6 |  | 2 | 3 | 0 |  | 1 |
|  |  |  | Dissemination rate (%) |  | 100 | 100 |  | 33.33 | 60.00 | 0 |  | 50 |
|  |  |  | CI95% (%) |  | 0-84.19* | 0-45.93* |  | 4.33-77.72 | 14.66-94.73 | 0-97.5* |  | 1.26-98.74 |
|  |  | Mutant | Survivor mosquitoes | 59 | 66 | 38 | 61 | 50 | 59 | 40 | 41 | 13 |
|  |  |  | PCR-positive | 7 | 0 | 5 | 32 | 31 | 40 | 4 | 17 | 11 |
|  |  |  | Dissemination rate (%) | 11.86 | 0 | 13.16 | 52.46 | 62 | 67.8 | 10 | 41.46 | 84.62 |
|  |  |  | CI95% (%) | 4.91-22.93 | 0-5.44* | 4.41-28.09 | 39.27-65.40 | 47.17-75.35 | 54.36-79.38 | 2.79-23.66 | 26.32-57.89 | 54.55-98.08 |

(*) one-sided, 95% confidence interval
